# Supplementary material for: Non-communicable disease policy implementation in Libya: A mixed methods assessment
Source: PLOS Glob Public Health. 2022 Nov 10;2(11):e0000615. doi: 10.1371/journal.pgph.0000615 (PMC10021530; doi:10.1371/journal.pgph.0000615)
Supplement: S1 Checklist — (DOCX) [file pgph.0000615.s001.docx]

**COREQ (Consolidated criteria for reporting qualitative research)**

**Domain 1: Research team and reflexivity**

*Personal characteristics*

1. Interviewer

CW, LA and MK conducted the interviews.

2. Credentials and 3. Occupation

Credentials and occupations have been listed in the manuscript.

4. Gender

LA – male

CW – female

GL – female

MK – female

MA – male

TE - ?

AB – female

AH – male

CH – male

HE - ?

5. Experience and training

LA is an experienced researcher in global health. CW has qualitative research experience. GL has extensive global health research experience. MK, AB and CH are senior health specialists at the World Bank with extensive experience in global health in the Middle East and Northern Africa. MA, TE and HE are senior public health officials in the Ministry of Health. AB

*Relationship with participants*

6. Relationship established

Relationships were established with potential participants through MK’s networks prior to the study.

7. Participant knowledge of the interviewer

The participants knew the reasons for conducting the research (detailed in the patient information and consent form), and participants were aware that the study would specifically ask about their experiences implementing NCD policy in Libya.

8. Interviewer characteristics

Participants knew of the interviewers interests in the research topic. Both LA and MK have significant experience in the area and

**Domain 2: Study design**

*Theoretical framework*

9. Methodological orientation and Theory

This applied health systems research was underpinned by a positivist approach, and utilised an applied framework analysis.

*Participant selection*

10. Sampling and 11. Method of approach

Participants were selected purposively for their key roles and diverse experiences within NCD policy-making in Libya. Participants were approached by MK who established their initial interest in participating in an interview.

12. Sample size

Five interviews were conducted with policymakers.

13. Non-participation

No participants declined to participate.

*Setting*

14. Setting of data collection

Interviews took place online via videocall.

15. Presence of non-participants

No non-participants were present.

16. Description of sample

Participants were key policymakers and NCD policy stakeholders that had been previously identified in a separate mapping exercise conducted by World Bank and MoH staff. Participants were senior members of government and international NGOs, including representatives from the WHO, the International Rescue Committee, the National Center for Disease Control (NCDC), and the Libyan Food and Drug Control Center (FDCC). All interviewees spoke fluent English and did not require translators.

*Data collection*

17. Interview guide

A semi-structured interview schedule was used. It was not pilot tested.

18. Repeat interviews

Repeat interviews were not conducted.

19. Audio recording

Interviews were audio recorded.

20. Field notes

Field notes were made during and after the interviews.

21. Duration

Interviews lasted approximately 50-60 minutes.

22. Data saturation

While further interviews may have strengthened the sample, it was not possible to recruit more participants at the time.

23. Transcripts returned

Transcripts were not returned to participants for checking.

**Domain 3: analysis and findings**

*Data analysis*

24. Number of data coders

Data was coded by CW and findings were peer-checked by LA and MK.

25. Description of the coding tree

A description of the coding tree is not provided.

26. Derivation of themes

Framework analysis was deductive and derived from the systematic review framework.

27. Software

NVivo software was used to manage the data.

28. Participant checking

Participants did not provide feedback on the findings.

*Reporting*

29. Quotations presented

Participant quotations are presented throughout the manuscript by general participant identifiers so as to protect the identity of participants.

30. Data and findings consistent

There was good consistency between data and findings, with the wider research team providing critique and consensus on findings.

31. and 32. Clarity of major and minor themes

Major themes are presented; there were no minor themes.
