# Supplementary material for: Non-communicable disease policy implementation in Libya: A mixed methods assessment
Source: PLOS Glob Public Health. 2022 Nov 10;2(11):e0000615. doi: 10.1371/journal.pgph.0000615 (PMC10021530; doi:10.1371/journal.pgph.0000615)
Supplement: S1 Text — (DOCX) [file pgph.0000615.s002.docx]

S1 Text: Appendix

### 1. Search Strategy and Search Terms (MEDLINE)

1 cardiovascular diseases/ or heart diseases/ or vascular diseases/ or cerebrovascular diseases/

2 exp myocardial ischemia/

3 Heart failure/

4 exp brain ischemia/ or exp stroke/

5 exp diabetes mellitus, type 2/

6 lung diseases, obstructive/ or exp pulmonary disease, chronic obstructive/

7 exp *neoplasms/

8 ((cardiovascular or cardio-vascular) adj3 disease*).ti,ab.

9 ((cardiovascular or cardio-vascular) adj3 (event* or outcome* or risk*)).ti,ab.

10 ((coronary or heart or myocard*) adj3 disease*).ti,ab.

11 ((coronary or heart or myocard*) adj3 (event* or outcome* or risk*)).ti,ab.

12 ((ischaemic or ischemic or ischaemia or ischemia) adj3 disease*).ti,ab.

13 ((ischaemic or ischemic or ischaemia or ischemia) adj3 (event* or outcome* or risk*)).ti,ab

14 myocardial infarct*.ti,ab.

15 ((cerebrovascular or vascular) adj3 disease*).ti,ab.

16 ((cerebrovascular or vascular) adj3 (event* or outcome* or risk*)).ti,ab.

17 stroke.ti,ab.

18 heart failure.ti,ab.

19 diabet*.ti.

20 ((type 2 or type ii or noninsulin dependent or non insulin dependent or adult onset or maturity onset or obes*) adj2 diabet*).ti,ab.

21 (niddm or t2dm or tiidm).ti,ab.

22 (chronic adj2 (lung or pulmonary)).ti,ab.

23 chronic obstructive pulmonary disease.ti,ab.

24 (neoplas* or cancer* or carcinoma* or tumor* or tumour* or malignan* or leukaemia or leukemia or lymphoma?).ti,ab.

25 1 or 2 or 3 or 4 or 5 or 6 or 7 or 8 or 9 or 10 or 12 or 13 or 14 or 15 or 16 or 17 or 18 or 19 or 20 or 21 or 22 or 23 or 24

26 taxes/ and ("tobacco use"/ or exp "tobacco use cessation"/ or drinking behavior/ or exp alcohol drinking/ or exp dietary fats/ or sodium chloride/)

27 (tax or taxes or taxing or taxation).ti.

28 ((food? or diet* or vegetable? or fruit? or sugar* or fat or fats or sucrose or candy or sweet* or snack* or fastfood? or junkfood?) and (tax or taxes or taxing or taxation)).ti,ab.

29 ((smok* or tobacco) and (tax or taxes or taxing or taxation)).ti,ab.

30 ((alcohol or drinking) and (tax or taxes or taxing or taxation)).ti,ab.

31 ((smok* or tobacco) and (subsidy or subsidies or incentiv* or voucher?)).ti,ab.

32 ((alcohol or drinking) and (subsidy or subsidies or incentiv* or voucher?)).ti,ab.

33 (social control, formal/ or legislation/) and ("tobacco use"/ or exp "tobacco use cessation"/ or tobacco industry/ or tobacco smoke pollution/ or drinking behavior/ or exp alcohol drinking/ or exp dietary fats/ or fast foods/ or sodium chloride/)

34 smoke-free policy/

35 exp nutrition policy/

36 ((smoke or smoking or tobacco) adj2 (ban or bans or banned or free)).ti,ab.

37 ((smoke or smoking or tobacco) and (legislat* or law? or regulation or regulatory)).ti,ab.

38 ((alcohol or drinking) and (legislat* or law? or regulation or regulatory)).ti,ab.

39 exp trans fatty acids/

40 social marketing/

41 (marketing/ or advertising as topic/ or mass media/ or product packaging/ or product labeling/) and ("tobacco use"/ or exp "tobacco use cessation"/ or drinking behavior/ or exp alcohol drinking/ or exp diet/ or food/ or exp dietary fats/ or fast foods/ or fruit/ or vegetables/ or sodium chloride, dietary/ or beverages/ or exp exercise/ or motor activity/ or health behavior/ or risk reduction behavior/)

42 food packaging/ or food labeling/

43 ((food? or diet* or vegetable? or fruit? or sugar* or fat or fats or sucrose or candy or sweet* or snack* or fastfood? or junkfood?) and (marketing or adverti?ing or sponsorship? or label* or pack*)).ti,ab.

44 ((smok* or tobacco) and (marketing or adverti?ing or sponsorship? or label* or pack*)).ti,ab.

45 exp alcohol drinking/ and retail.mp

46 exp alcohol drinking/ and restriction.mp

47 ((alcohol drinking) and (marketing or adverti?ing or sponsorship? or label* or pack*)).ti,ab.

48 (salt adj3 (intake or reduc* or lower*)).ti,ab.

49 health promotion/ and (neoplasms/ or obesity/ or diabetes mellitus, type 2/ or smoking/ or risk factors/ or cardiovascular diseases/ or hypertension/)

50 drug therapy, combination and (diabetes mellitus, type 2/ or cardiovascular diseases/ or hypertension/)

51 patient education as topic/ or counseling/ or patient compliance/ or motivational interviewing/ and (neoplasms/ or obesity/ or diabetes mellitus, type 2/ or smoking/ or risk factors/ or cardiovascular diseases/ or hypertension/)

52 health education/ or health knowledge, attitudes, practice/ and (life style/ or food habits/ or diet/ or health behavior/ or obesity/ or motor activity/

53 hydroxymethylglutaryl-coa reductase inhibitors/ or simvastatin/ or aspirin/ or metformin/ or adrenergic beta-antagonists/ or propranolol/

54 food legislation/ and (dietary fats/ or sodium/)

55 food supply/ and (dietary fats/ or sodium/)

56 Papanicolaou test/

57 mass screening/ and (uterine cervical neoplasms/ or cervical intraepithelial neoplasia/)

58 ((cervical or pap) adj3 screen*).ti,ab.

59 hepatitis b vaccines/

60 ((hepatitis b or hep b) adj3 (vaccin* or immuni?ation or immuni?e)).ti,ab.

61 *aspirin/

62 primary prevention/ and aspirin/

63 (aspirin or acetylsalicylic acid).ti,ab.

64 best buys.mp

65 or/26-64

66 25 and 65

### 2. Inclusion and Exclusion Criteria for Full Texts

| **Inclusion criteria** | **Include** | **Exclude** |
| --- | --- | --- |
| Population and setting | Any population where non-communicable disease policy was developed |  |
| Intervention | Involve a policy or intervention in line with the World Health Organization Best Buys | Studies that examined only one portion of the population or individual-level approach, behavioral change |
| Outcome | Barriers to or facilitators of adoption and implementation from a political economy perspective. May include organizational outcomes, process outcomes, funding, legal frameworks for implementation (or lack thereof), communities/demand side | Cost effectiveness only; not reporting outcomes under study |
| Study design | Primary or empirical research; may be randomized controlled trials or non-randomized controlled trials, natural experiments, mixed-methods studies, policy analysis, key informant interviews, policy documents on non-communicable disease prevention (e.g., acts and laws, strategic plans, guidelines, government directives), case studies in successful policy formulation and implementation at national or international level | Case-control studies, clinical studies, behavioral, etiological, systematic, and scoping reviews, literature reviews |

### 3. Data Extraction Form

| **Variables names** | **Comment** |
| --- | --- |
| **Paper N** | ID number assigned to included paper list |
| **First author** | First author |
| **Year** | Year of publication |
| **Link** | Link to online article |
| **Title** | Full title of the article |
| **Author affiliation** | Affiliation of first author |
| **Country of study** | Where study was conducted |
| **Setting definition** | HIC, MIC, LIC, global (if multi-countries study across HIC and LMIC); |
| **Aim of study** | The aim of the study as usually stated in the methods or introduction |
| **Dates of study** | Time period of the study or when research was conducted |
| **Methodology** | See methods section of paper, Please select from  Primary: qualitative; Primary: quantitative Primary: mixed-method Secondary: document analysis, etc; |
| **Quality appraisal** | As per MMAT -separate excel matrix |
| **NCD policy area** | Specify the original Best Buy policy (based on protocol) or NCD policy area (eg, tobacco control, PA education) |
| **NCD policy definition used** | Policy mentioned in the paper and how it is adopted or implemented |
| **National policy mentioned** | Report national policy name |
| **Global policy mentioned** | Report global policy name (eg, WHO FCTC) |
| **Implementation or adoption** | Please state if article is about implementation or adoption or other (and specify). Policy adoption is the third phase of the policy process in which policies are adopted by government bodies for future implementation. Policy implementation is the fourth phase of the policy cycle in which adopted policies are put into effect. |
| **Implementation or adoption status** | Are these studies focused on when Best Buys are adapted and discussed, when they are rolled out, when they are fully being implemented and reviewed. Please select: Partially implemented, fully implemented, fully adopted, partially adopted, Rolled out, Reviewed |
| **Implementation step** | Step 1: Identification of appropriate policy or intervention; Step 2: Adaptation and piloting of policy and intervention; Step 3: Evaluation of the implementation of policy or intervention; Step 4: Scale up of policy or intervention |
| **Actors** | State stakeholders involved. Please clearly state details as reported. |
| **Actors 3Is** | 3I+N framework that includes: interests, ideas, institutions and networks (if available, if not specify Not Reported=NR) |
| **Implementation or adoption level** | Please specify: Global, Regional or multi-country, National, Sub-national (eg, district, provincial) |
| **Barriers/challenges** | Add quotes from article on barriers and challenges mentioned (if not mentioned, please say NR=Not reported). Add quotes (eg, from interviews) and/or entire extracts where relevant |
| **Opportunities/facilitators** | Add quotes from article on solutions and opportunities mentioned or Not Reported=NR or Unclear. Add quotes (eg, from interviews) and/or entire extracts where relevant |
| **Contextual features** | Overview of the regions and contextual features as reported (add extracts or quotes) |
| **Comments** | Any additional comment |

### 4. PRISMA diagram

Full-text articles excluded

(n = 580)

*Lacking policy process focus on adoption or implementation; n = 251*

*Lacking Best Buys measure specificity; n = 112*

*Study type (review, commentary, etc); n= 196*

*Other (language, article not available); n = 21*

Records excluded
(n = 8,652)

Studies included in qualitative synthesis
(n = 186)

Additional records identified through other sources
(n = 163)

Records identified through database searching
(n = 9496)

Titles screened
(n = 9418)

Full-text articles assessed for eligibility
(n = 766)

Duplicates removed
(n = 241)

**Eligibility**

**Identification**

**Screening**

**Included**

### **5. Risk of Bias Assessment**

Confidence in Evidence from Reviews of Qualitative research (CERQual) Summary of Qualitative Findings

| **Summary of review findings** | **Studies contributing to review finding (see list below for references)** | **CERQual assessment of confidence in the evidence** | **Explanation of CERQual assessment** |
| --- | --- | --- | --- |
| **Gather data** | | | |
| Countries with limited resources or lack of NCD plans are advised to make use of existing surveillance systems and structures. | 75, 81, 86, 173 | **Moderate** | Minor concerns regarding methodological limitations, relevance, coherence; moderate concerns regarding adequacy (because of limited data) |
| **Convene cross-sectoral NCD committee** | | | |
| Multi-stakeholder committees should be established at national and subnational levels with clear mandate and scope (and adequate resources). | 12, 68, 71, 73, 75, 168, 175, 55, 60, 61, 65, 69, 82, 83, 84, 85, 151, 24, 27, 30, 31, 33, 54, 97, 100, 104, 105, 107, 111, 154, 163, 164, 183 | **High** | Minor methodological limitations; data from several countries providing a relatively well-generalizable finding; no or very minor concerns regarding coherence and adequacy |
| Multi-stakeholder committees need a clear accountability framework regulated by process and outcome indicators. | 2, 3, 4, 6, 7, 9, 10, 94 115, 117, 118, 145, 146 | **High** | Minor methodological limitations; coherent, relevant, adequate data from studies |
| Multi-stakeholder committees should include researchers, community-based groups, nongovernmental organizations, civil society, clinicians, patient organizations. | 2, 5, 6, 8, 9, 10, 20, 23 30, 31, 35, 37, 41, 46, 51,109 | **High** | Minor methodological limitations; no or very minor concerns regarding adequacy, relevance, and coherence |
| Private sector can inform governments, but must be without conflict of interest and not able to influence policy decisions via multi-stakeholder committee. | 2, 55, 56, 57, 58, 67, 72, 75, 82, 119, 123, 145, 123, 124, 143, 145, 115, 117, 118, 143, 145, 146 | **High** | Minor methodological limitations; no or very minor concerns regarding adequacy, relevance, and coherence |
| **Tobacco** | | | |
| Use Framework Convention for Tobacco Control instruments and MPOWER package to develop mass media campaigns; seek support of civil society and other allies and champions. | 67, 69, 86, 115, 118, 123, 134, 136, 141, 142, 143, 145 | **High** | Minor methodological limitations; minor or moderate concerns regarding adequacy and relevance of data; no concerns regarding coherence |
| Increase prices by imposing excise taxes on imports of raw tobacco and increasing excise taxes on finished tobacco products. | 125, 127, 129, 130, 139, 140, 141 | **High** | Minor or no concerns regarding methodological limitations; minor or moderate concerns regarding relevance; minor concerns regarding adequacy; some concerns regarding fit between data from primary data and review findings |
| **Physical activity** | | | |
| Adopt multi-sectoral collaboration and engage ministries and departments of sport, health, education, recreation and leisure, research, transport, urban and rural planning, and design to promote physical activity; use World Health Organization materials for national campaigns. | 90, 92, 93, 94, 95, 96, 166 | **Moderate** | Minor or no concerns regarding methodological limitations; minor or moderate concerns regarding relevance; moderate concerns regarding adequacy; no concerns regarding coherence |
| **Diet** | | | |
| Develop healthy diet coalition (multi-sectoral subcommittee of national non-communicable disease committee) with government stakeholders (e.g., Ministry of Agriculture, Food and Drug Control Center). | 35, 45, 51, 154, 163, 35, 43, 45, 51, 24, 27, 30, 31, 33, 54, 97, 100, 103, 104, 105, 107, 111, 154, 163, 164, 183 | **High** | Minor methodological limitations; minor or moderate concerns regarding adequacy and relevance of data; no concerns regarding coherence |
| Set thresholds within constraints of current trade agreements. | 26, 29, 30, 31, 40, 49, 50, 54, 98, 100, 101, 104, 167, 183 | **High** | Minor or no methodological limitations; minor concerns regarding coherence, adequacy; minor or moderate concerns regarding relevance |
| Introduce mandatory labeling of salt, trans and saturated fats, and sugars. | 25, 26, 36, 37, 39, 49, 50, 54, 98, 99 | **High** | Minor or no methodological limitations; minor concerns regarding coherence and adequacy; minor or moderate concerns regarding relevance |
| **Clinical guidelines** | | | |
| Work with primary health care institutes to disseminate national guidelines and standards. | 151, 154, 174, 179, 183 | **Moderate** | Minor methodological limitations; minor concerns regarding coherence; moderate concerns regarding adequacy; moderate concerns regarding relevance |

### 6. List of included studies

| **No.** | **Determin-ant of health** | **Reference** | **Country of study** | **Context classification** | **Method** |
| --- | --- | --- | --- | --- | --- |
| 1 | Alcohol | [Opeyemi Abiona, Mojisola Oluwasanu, and Oladimeji Oladepo. 2019. “Analysis of Alcohol Policy in Nigeria: Multi-Sectoral Action and the Integration of the WHO “Best-Buy” Interventions.” BMC Public Health 19: 810. https://doi.org/10.1186/s12889-019-7139-9](https://doi.org/10.1186/s12889-019-7139-9) | Nigeria | Fragile | Mixed method |
| 2 | Alcohol | [Colin Angus, John Holmes, and Petra S Meier. 2019. “Comparing Alcohol Taxation Throughout the European Union.” Addiction 114: 1489–1494. https://doi.org/10.1111/add.14631.](https://doi.org/10.1111/add.14631) | 28 EU Member | Non-fragile (HIC + MIC) | Descriptive analysis |
| 3 | Alcohol | [David A. Keatley, Sarah J. Hardcastle, Natacha Carragher, Tanya N. Chikritzhs, Mike Daube, Adam Lonsdale, and Martin S. Hagger. 2018. “Attitudes and Beliefs Towards Alcohol Minimum Pricing in Western Australia.” Health Promotion International 33 (3): 400–409. https://doi.org/10.1093/heapro/daw092](https://doi.org/10.1093/heapro/daw092) | Australia | Non-fragile (HIC) | Policy analysis |
| 4 | Alcohol | Mary Lawhon and Clare Herrick. 2013. “Alcohol Control in the News: The Politics of Media Representations of Alcohol Policy in South Africa.” *Journal of Health Politics, Policy, and Law* 38 (5): 987–1021. doi: 10.1215/03616878-2334683. Epub 2013 Jun 21. PMID: 23794743. | South Africa | Non-fragile (UMIC) | Document analysis |
| 5 | Alcohol | [Jessica Li, Melanie Lovatt, Douglas Eadie, Fiona Dobbie, Petra Meier, John Holmes, Gerard Hastings, and Anne Marie MacKintosh. 2017. “Public Attitudes Towards Alcohol Control Policies in Scotland and England: Results from a Mixed-Methods Study.” Social Science and Medicine 177: 177–189. https://doi.org/10.1016/j.socscimed.2017.01.037](https://doi.org/10.1016/j.socscimed.2017.01.037) | UK | Non-fragile (HIC) | Mixed method |
| 6 | Alcohol | [Rupali J. Limaye, Lainie Rutkow, Rajiv N. Rimal, and David H Jernigan. 2014. “Informal Alcohol in Malawi: Stakeholder Perceptions and Policy Recommendations.” Journal of Public Health Policy 35: 119–131. https://doi.org/10.1057/jphp.2013.43](https://doi.org/10.1057/jphp.2013.43) | Malawi | Non-fragile (LIC) | Qualitative method |
| 7 | Alcohol | [Beatrice L. Matanje Mwagomba, Misheck J. Nkhata, Alex Baldacchino, Jennifer Wisdom, and Bagrey Ngwira. 2018. “Alcohol Policies in Malawi: Inclusion of WHO “Best Buy” Interventions and Use of Multi-Sectoral Action.” BMC Public Health 18: 957. https://doi.org/10.1186/s12889-018-5833-7](https://doi.org/10.1186/s12889-018-5833-7) | Malawi | Non-fragile (LIC) | Case study |
| 8 | Alcohol | Eva Jané-Llopis, Daša Kokole, Maria Neufeld, Omer Syed Muhammad Hasan, and Jürgen Rehm. 2020. “What Is the Current Alcohol Labelling Practice in the WHO European Region and What Are Barriers and Facilitators to Development and Implementation of Alcohol Labelling Policy?” Health Evidence Network Synthesis Report No. 68. Copenhagen: WHO Regional Office for Europe. | Europe | Non fragile (HIC) | Case study |
| 9 | Alcohol | [Kidong Park. 2019. “New Law on Prevention and Control of Alcohol Related Harms in Vietnam.” Journal of Global Health Science 1 (2): e49. https://doi.org/10.35500/jghs.2019.1.e49](https://doi.org/10.35500/jghs.2019.1.e49) | Vietnam | Non-fragile (LMIC) | Qualitative method |
| 10 | Alcohol | Dag Rekve, Nicholas Banatvala, Adam Karpati, Dudley Tarlton, Lucinda Westerman, Kristina Sperkova, Sally Casswel, Maik Duennbie, Ariella Rojhani, Øystein Bakke , Maristela Monteiro, Natalia Linou, Alexey Kulikov, and Vladimir B Poznyak. 2019. “Prioritising Action on Alcohol for Health and Development.” *BMJ* 367: l6162. doi:10.1136/bmj.l6162 | Global | Global | Policy analysis |
| 11 | Alcohol | [Karen Wallace and Bayard Roberts. 2014. “An Exploration of the Alcohol Policy Environment in Post-Conflict Countries.” Alcohol and Alcoholism 49 (3): 356–362: https://doi.org/10.1093/alcalc/agt142](https://doi.org/10.1093/alcalc/agt142) | conflict affected states | Fragile | Case study |
| 12 | Alcohol, diet, tobacco, physical activity | [Vivian Lin and Bronwyn Carter. 2013. “From Healthy Public Policy to Intersectoral Action and Health-in-All Policies.” In Global Handbook on Noncommunicable Diseases and Health Promotion, edited by D. McQueen, 189–201. New York: Springer. https://doi.org/10.1007/978-1-4614-7594-1_12](https://doi.org/10.1007/978-1-4614-7594-1_12) | Global case studies | Global | Mixed method |
| 13 | Cancer | [Ranajit Mandal and Partha Basu. 2018. “Cancer Screening and Early Diagnosis in Low and Middle Income Countries.” Bundesgesundheitsbl 61: 1505–1512. https://doi.org/10.1007/s00103-018-2833-9](https://doi.org/10.1007/s00103-018-2833-9) | LMICs | Global | Document analysis |
| 14 | Cancer | [Malcolm A. Moore. 2014. “Cancer Control Programs in East Asia: Evidence from the International Literature.” Journal of Preventative Medicine and Public Health 47 (4). https://doi.org/10.3961/jpmph.2014.47.4.183](https://doi.org/10.3961/jpmph.2014.47.4.183) | East Asia | Non fragile (HIC+MIC) | Case study |
| 15 | Cancer | [Justin O. Parkhurst and Madhulika Vulimiri. 2013. “Cervical Cancer and the Global Health Agenda: Insights from Multiple Policy-Analysis Frameworks.” Global Public Health 8 (10): 1093–1108. doi: 10.1080/17441692.2013.850524](https://doi.org/10.1080/17441692.2013.850524) | Global | Global | Policy analysis |
| 16 | Cancer | Diana Sarfati, Rachel Dyer, Filipina Amosa-Lei Sam, et al. 2019. “Cancer Control in the Pacific: Big Challenges Facing Small Island States.” *Lancet Oncology* 20 (9): e475–e492. | Pacific island countries and territories (PICTs) | Fragile (MIC) [not for OECD] | Case study |
| 17 | Cancer | Dingle Spence, Rachel Dyer, Glennis Andall-Brereton, et al 2019. “Cancer Control in the Caribbean Island Countries and Territories: Some Progress but the Journey Continues.” *Lancet Oncology* 20 (9): e503–e521. | Carribean Island countries | Non-fragile (MIC) | Case study |
| 18 | Cancer | Silvina Frech, Catherine A. Muha, Lisa M. Stevens, et al 2018. “Perspectives on Strengthening Cancer Research and Control in Latin America Through Partnerships and Diplomacy: Experience of the National Cancer Institute's Center for Global Health.” *Journal of Global Oncology* 4: 1-11. doi:10.1200/JGO.17.00149 | Latin America | Non fragile (MIC) | Case study |
| 19 | Diabetes | [Willemijn E. De Bruin, Cherie Stayner, Michel de Lange, and Rachael W. Taylor. 2018. “Who Are the Key Players Involved with Shaping Public Opinion and Policies on Obesity and Diabetes in New Zealand?” Nutrients 10 (11): 1592. https://doi.org/10.3390/nu10111592](https://doi.org/10.3390/nu10111592) | New Zealand | Non-fragile (HIC) | Social Network analysis |
| 20 | Diabetes | [Tint Swe Latt, Than Than Aye, Ko Ko, and Ko Ko Zaw. 2016. “Gaps and Challenges to Integrating Diabetes Care in Myanmar.” WHO South-East Asia Journal of Public Health 5 (1): 48–52. https://doi.org/10.4103/2224-3151.206553](https://doi.org/10.4103/2224-3151.206553) | Myanmar | Fragile (LMIC) | Document analysis |
| 21 | Diabetes | Linda Penn, Angela Rodrigues, Anna Haste, Marta M Marques, Kirsten Budig, Kirby Sainsbury, Ruth Bell, Vera Araújo-Soares, Martin White, Carolyn Summerbell, Elizabeth Goyder, Alan Brennan, Ashley J Adamson, and Falko F Sniehotta. 2018. “NHS Diabetes Prevention Programme in England: Formative Evaluation of the Programme in Early Phase Implementation.” *BMJ Open* 8: e019467. doi: 10.1136/bmjopen-2017-019467 | England | Non-fragile (HIC) | Mixed method |
| 22 | Diabetes | André M. N. Renzaho. 2015. “The Post-2015 Development Agenda for Diabetes in Sub-Saharan Africa: Challenges and Future Directions.” *Global Health Action* 8: 27600. https://doi.org/10.3402/gha.v8.27600 | Sub-Saharan Africa | Non-fragile (UMIC) | Case study |
| 23 | Diabetes | [Veronica Shiroya, Florian Neuhann, Olaf Müller, and Andreas Deckert. 2019. “Challenges in Policy Reforms for Non-Communicable Diseases: The Case of Diabetes in Kenya.” Global Health Action 12: 1. doi: 10.1080/16549716.2019.1611243](https://doi.org/10.1080/16549716.2019.1611243) | Kenya | Fragile (LMIC) | Document analysis + interviews |
| 24 | Diet | [Joao Breda, Lea Samant, Nash Castro, Stephen Whiting, Julianne Williams, Jo Jewell, Kaia Engesveen, and Kremlin Wickramasinghe. 2020. “Towards Better Nutrition in Europe: Evaluating Progress and Defining Future Directions.” Food Policy 96: 101887. https://doi.org/10.1016/j.foodpol.2020.101887](https://doi.org/10.1016/j.foodpol.2020.101887) | Member States in the WHO European Region | Non-fragile (HIC + MIC) | Survey |
| 25 | Diet | [Tracy Comans, Nicole Moretto. and Joshua Byrnes. 2017. “Public Preferences for the Use of Taxation and Labelling Policy Measures to Combat Obesity in Young Children in Australia.” International Journal of Environmental Research and Public Health 14 (3): 324. https://doi.org/10.3390/ijerph14030324](https://doi.org/10.3390/ijerph14030324) | Australia | Non-fragile (HIC) | Survey |
| 26 | Diet | [A. Gesser-Edelsburg, R. Endevelt, and Y. Tirosh-Kamienchick. 2014. Nutrition Labelling and the Choices Logo in Israel: Positions and Perceptions of Leading Health Policy Makers. Journal of Human Nutrition and Dietetics: The Official Journal of the British Dietetic Association 27 (1): 58–68. https://doi.org/10.1111/jhn.12050](https://doi.org/10.1111/jhn.12050) | Israel | Non-fragile (HIC) | Qualitative method |
| 27 | Diet | [Melissa Mialon, Jonathan Mialon, Giovanna Calixto Andrade, and Moubarac Jean-Claude. 2020. “‘We Must Have a Sufficient Level of Profitability’: Food Industry Submissions to the French Parliamentary Inquiry on Industrial Food.” Critical Public Health 30 (4): 457–467. doi: 10.1080/09581596.2019.1606418](https://doi.org/10.1080/09581596.2019.1606418) | France | Non-fragile (HIC) | Document analysis |
| 28 | Diet | [Melissa Mialon, Boyd Swinburn, Steven Allender, and Gary Sacks. 2016. “Systematic Examination of Publicly-Available Information Reveals the Diverse and Extensive Corporate Political Activity of the Food Industry in Australia.” BMC Public Health 16: 283. https://doi.org/10.1186/s12889-016-2955-7](https://doi.org/10.1186/s12889-016-2955-7) | Australia, UK | Non-fragile (HIC) | Case study |
| 29 | Diet | [Melissa Mialon, Boyd Swinburn, Jillian Wate, Isimeli Tukana, and Gary Sacks. 2016. “Analysis of the Corporate Political Activity of Major Food Industry Actors in Fiji.” Global Health 12: 18. https://doi.org/10.1186/s12992-016-0158-8](https://doi.org/10.1186/s12992-016-0158-8) | Fiji | Non-fragile (UMIC) | Mixed method |
| 30 | Diet | [R. Pérez-Escamilla, C.K. Lutter, C. Rabadan-Diehl, A. Rubinstein, A. Calvillo, C. Corvalán, C. Batis, E. Jacoby, S. Vorkoper, L. Kline, E. Ewart-Pierce, and J.A. Rivera. 2017. “Prevention of Childhood Obesity and Food Policies in Latin America: From Research to Practice.” Obesity Reviews : An Official Journal of the International Association for the Study of Obesity 18 Suppl 2: 28–38. https://doi.org/10.1111/obr.12574](https://doi.org/10.1111/obr.12574) | Mexico, Chile, Ecuadaor, Argentina | Non-fragile (UMIC) | Case study |
| 31 | Diet | [Tarryn Phillips, Amerita Ravuvu, Celia McMichael, Anne Marie Thow, Jennifer Browne, Gade Waqa, Jillian Tutuo, and Deborah Gleeson. 2021. “Nutrition Policy-Making in Fiji: Working in and Around Neoliberalisation in the Global South.” Critical Public Health 31 (3): 316–326. doi: 10.1080/09581596.2019.1680805](https://doi.org/10.1080/09581596.2019.1680805) | Fiji | Non-fragile (UMIC) | Mixed method |
| 32 | Diet | Jennifer L Pomeranz, Leslie Zellers, Michael Bare, and Mark Pertschuk. 2019. “State Preemption of Food and Nutrition Policies and Litigation: Undermining Government's Role in Public Health.” *American Journal of Preventive Medicine* 56 (1): 47–57. | USA | Non-fragile (HIC) | Policy analysis |
| 33 | Diet | [Erica Reeve, Anne Marie Thow, Colin Bell, Katrin Engelhardt, Ella Cecilia Gamolo-Naliponguit, John Juliard Go, and Gary Sacks. 2018. “Implementation Lessons for School Food Policies and Marketing Restrictions in the Philippines: A Qualitative Policy Analysis.” Global Health 14: 8. https://doi.org/10.1186/s12992-017-0320-y](https://doi.org/10.1186/s12992-017-0320-y) | Philippines | Non-fragile (UMIC) | Qualitative method |
| 34 | Diet | [C. Scott, B. Hawkins, and C. Knai. 2017.” Food and Beverage Product Reformulation as a Corporate Political Strategy.” Social Science and Medicine 172: 37–45. https://doi.org/10.1016/j.socscimed.2016.11.020](https://doi.org/10.1016/j.socscimed.2016.11.020) | USA | Non fragile (HIC) | Policy analysis |
| 35 | Diet | [B. Winburn. 2013. “Monitoring and Benchmarking Government Policies and Actions to Improve the Healthiness of Food Environments: A Proposed Government Healthy Food Environment Policy Index.” Obesity Reviews: An Official Journal of the International Association for the Study of Obesity 14 Suppl 1: 24–37. https://doi.org/10.1111/obr.12073](https://doi.org/10.1111/obr.12073) | Global | Global | Mixed method |
| 36 | Diet | [Anne Marie Thow, Alexandra Jones, Corinna Hawkes, Iqra Ali, and Ronald Labonté. 2018. “Nutrition Labelling Is a Trade Policy Issue: Lessons from an Analysis of Specific Trade Concerns at the World Trade Organization.” Health Promotion International 33 (4): 561–571. https://doi.org/10.1093/heapro/daw109](https://doi.org/10.1093/heapro/daw109) | Chile, Indonesia, Peru, Ecuador. Thailand | Non-fragile (MIC) | Document analysis |
| 37 | Diet | [Anne Marie Thow Alexandra Jones, Carmen Huckel Schneider, and Ronald Labonté. 2019. “Global Governance of Front-of-Pack Nutrition Labelling: A Qualitative Analysis.” Nutrients 11 (2): 268. https://doi.org/10.3390/nu11020268](https://doi.org/10.3390/nu11020268) | global | Global | Qualitative method |
| 38 | Diet | [Gade Waqa, Marj Moodie, Wendy Snowdon, Catherine Latu, Jeremaia Coriakula, Steven Allender, and Colin Bell. 2017. “Exploring the Dynamics of Food-Related Policymaking Processes and Evidence Use in Fiji Using Systems Thinking.” Health Research and Policy Systems 15: 74. https://doi.org/10.1186/s12961-017-0240-6](https://doi.org/10.1186/s12961-017-0240-6) | Fiji | Non-fragile (UMIC) | Qualitative method |
| 39 | Diet | Adrián Alberto Díaz, Paula Mariana Veliz, Gabriela Rivas-Mariño, Carina Vance Mafla, Luz María Martínez Altamirano, Cecilia Vaca Jones. 2017. “Etiquetado de Alimentos en Ecuador: Implementación, Resultados y Acciones Pendientes.” *Revista Panamericana de Salud Pública* 41: e54. | Ecuador | Non-fragile (MIC) | Case study |
| 40 | Diet | [Lainie Rutkow, Jesse Jones-Smith, Hannah J. Walters, Marguerite O’Hara, and Sara N. Bleich. 2016. “Factors That Encourage and Discourage Policy-Making to Prevent Childhood Obesity: Experience in the United States.” Journal of Public Health Policy 37: 514–527. https://doi.org/10.1057/s41271-016-0035-y](https://doi.org/10.1057/s41271-016-0035-y) | USA | Non-fragile (HIC) | Primary qualitative: KIIs |
| 41 | Diet | Sirinya Phulkerd, Stefanie Vandevijvere, Mark Lawrence, Viroj Tangcharoensathien, and Gary Sacks. 2017. “Level of Implementation of Best Practice Policies for Creating Healthy Food Environments: Assessment by State and Non-State Actors in Thailand.” *Public Health Nutrition* 20 (3): 381–390. doi:10.1017/S1368980016002391 | Thailand | Non fragile (MIC) | Mixed method |
| 42 | Diet | Donley Studlar and Paul Cairney. 2019. “Multilevel Governance, Public Health and the Regulation of Food: Is Tobacco Control Policy a Model?” *Journal of Public Health Policy* 40: 147–165. https://doi.org/10.1057/s41271-019-00165-6 | UK, USA | Non-fragile (HIC) | Document analysis |
| 43 | Diet | [Anne Marie Thow, Wendy Snowdon, Ronald Labonté, Deborah Gleeson, David Stuckler, Libby Hattersley, Ashley Schram, Adrian Kay, and Sharon Frielf. 2015. “Will the Next Generation of Preferential Trade and Investment Agreements Undermine Prevention of Noncommunicable Diseases? A Prospective Policy Analysis of the Trans Pacific Partnership Agreement.” Health Policy 119 (1): 88–96. https://doi.org/10.1016/j.healthpol.2014.08.002](https://doi.org/10.1016/j.healthpol.2014.08.002) | countries involved in trans-Pacific Partner- ship Agreement (TPPA) | Global | Policy analysis |
| 44 | Diet | Barbara Von Tigerstrom. 2013. “How Do International Trade Obligations Affect Policy Options for Obesity Prevention? Lessons from Recent Developments in Trade and Tobacco Control.” *Canadian Journal of Diabetes* 37 (3): 182–188 | WTO | Global | Document analysis |
| 45 | Diet | Boyd Swinburn, Vivica Kraak, Harry Rutter, Stefanie Vandevijvere, Tim Lobstein, Gary Sacks, Fabio Gomes, Tim Marsh, and Roger Magnusson. 2015. “Strengthening of Accountability Systems to Create Healthy Food Environments and Reduce Global Obesity.” *Lancet* 385 9986.: 2534–2545. | Global | Global | Case study |
| 46 | Diet | Dariush Mozaffarian, Sonia Y. Angell, Tim Lang, and Juan A. Rivera. 2018. “Role of Government Policy in Nutrition—Barriers to and Opportunities for Healthier Eating.” *BMJ* 361: k2426. doi:10.1136/bmj.k2426 | global | Global | Policy analysis |
| 47 | Diet | [Anne Marie Thow, David Sanders, Eliza Drury, Thandi Puoane, Syeda N. Chowdhury, Lungiswa Tsolekile, and Joel Negin. 2015. “Regional Trade and the Nutrition Transition: Opportunities to Strengthen NCD Prevention Policy in the Southern African Development Community.” Global Health Action 8: 1. doi: 10.3402/gha.v8.28338](https://doi.org/10.3402/gha.v8.28338) | 15 countries in Southern African Development Community (SADC) | Non-fragile (LMIC) | Policy analysis |
| 48 | Diet, tobacco | [R. S. Magnusson. 2015. “Case Studies in Nanny State Name-Calling: What Can We Learn?” Public Health 129 (8): 1074–1082. https://doi.org/10.1016/j.puhe.2015.04.023](https://doi.org/10.1016/j.puhe.2015.04.023) | Global | Global | Case study |
| 49 | Fat | [Uriyoán Colón-Ramos, Rafael Monge-Rojas, and Hannia Campos. 2014. “Impact of WHO Recommendations to Eliminate Industrial Trans-Fatty Acids from the food supply in Latin America and the Caribbean.” Health Policy and Planning 29 (5): 529–541. https://doi.org/10.1093/heapol/czt034](https://doi.org/10.1093/heapol/czt034) | 13 countries in Latin America and the Caribbean (Argentina, Brazil, | Non-fragile (MIC) | Mixed method |
| 50 | Fat | [Shauna M. Downs, Anne Marie Thow, Suparna Ghosh-Jerath, and Stephen R. Leeder. 2015. “Aligning Food-Processing Policies to Promote Healthier Fat Consumption in India.” Health Promotion International 30 (3): 595–605. https://doi.org/10.1093/heapro/dat094](https://doi.org/10.1093/heapro/dat094) | India | Non-fragile (MIC) | Mixed method |
| 51 | Fat | [World Health Organization. ‎2019‎. Countdown to 2023: WHO Report on Global Trans-Fat Elimination 2019. Washington, DC: World Health Organization. https://apps.who.int/iris/handle/10665/331300.](https://apps.who.int/iris/handle/10665/331300) | global | Global | Case study |
| 52 | Hypertension, diabetes | [Meena Daivadanam , Maia Ingram, Kristi Sidney Annerstedt, et al, on behalf of the GACD Concepts and Contexts working group. 2019. “The Role of Context in Implementation Research for Non-Communicable Diseases: Answering the ‘How-To’ Dilemma.” PLoS ONE 14 (4): e0214454. https://doi.org/10.1371/journal.pone.0214454](file:///C:\Users\User\Desktop\Libya%20Best%20Buys\Meena%20Daivadanam%20,%20Maia%20Ingram,%20Kristi%20Sidney%20Annerstedt,%20et%20al,%20on%20behalf%20of%20the%20GACD%20Concepts%20and%20Contexts%20working%20group.%202019) | Global | Global | Survey |
| 53 | Hypertension, diet | [Amos K. Laar, Alma J. Adler, Agnes M. Kotoh, Helena Legido-Quigley, Isabelle L. Lange, Pablo Perel, and Peter Lamptey. 2019. “Health System Challenges to Hypertension and Related Non-Communicable Diseases Prevention and Treatment: Perspectives from Ghanaian Stakeholders.” BMC Health Services Research 19: 693. https://doi.org/10.1186/s12913-019-4571-6](https://doi.org/10.1186/s12913-019-4571-6) | Ghana | Non-fragile (LMIC) | Qualitative method |
| 54 | Hypertension, diet | [Roger Magnusson and Belinda Reeve. 2015. “Food Reformulation, Responsive Regulation, and "Regulatory Scaffolding": Strengthening Performance of Salt Reduction Programs in Australia and the United Kingdom.” Nutrients 7 (7): 5281–5308. https://doi.org/10.3390/nu7075221](https://doi.org/10.3390/nu7075221) | Australia, UK | Non-fragile (HIC) | Document analysis |
| 55 | NCD | [Salim M. Adib. 2014. Non Communicable Disease Prevention and Control Plan (NCD-PCP) for Lebanon. WHO Lebanon Office. https://extranet.who.int/ncdccs/Data/LBN_B3_Final%20plan%202014.pdf](https://extranet.who.int/ncdccs/Data/LBN_B3_Final%20plan%202014.pdf) | Lebanon | Non-fragile (MIC) | Case study |
| 56 | NCD | Luke N. Allen, Brian D. Nicholson, Beatrice Y.T. Yeung, and Francisco Goiana-da-Silva. 2020. “Implementation of Non-Communicable Disease Policies: A Geopolitical Analysis of 151 Countries.” *Lancet Global Health* 8 (1): e50–e58. | Analysis of 151 countries | Global | Geopolitical analysis |
| 57 | NCD | [Pepita Barlow, Ronald Labonte, Martin McKee, and David Stuckler. 2018. “Trade Challenges at the World Trade Organization to National Noncommunicable Disease Prevention Policies: A Thematic Document Analysis of Trade and Health Policy Space.” PLoS Medicine 15 (6): e1002590. https://doi.org/10.1371/journal.pmed.1002590](https://doi.org/10.1371/journal.pmed.1002590) | 122 WTO member states | Global | Document analysis |
| 58 | NCD | [Tuhin Biswas, Sonia Pervin, Md. Imtiaz Alam Tanim, Louis Niessen, and Anwar Islam. 2017. “Bangladesh Policy on Prevention and Control of Non-Communicable Diseases: A Policy Analysis.” BMC Public Health 17: 582. https://doi.org/10.1186/s12889-017-4494-2](https://doi.org/10.1186/s12889-017-4494-2) | Bangladesh | Fragile (LMIC) | Policy analysis |
| 59 | NCD | [Elizabeth L. Budd, Anna J. deRuyter, Zhaoxin Wang, Pauline Sung-Chan, Xiangji Ying, Karishma S. Furtado, Tahna Pettman, Rebecca Armstrong, Rodrigo S. Reis, Jianwei Shi, Tabitha Mui, Tahnee Saunders, Leonardo Becker, and Ross C. Brownson. 2018. “A Qualitative Exploration of Contextual Factors that Influence Dissemination and Implementation of Evidence-Based Chronic Disease Prevention Across Four Countries.” BMC Health Services Research 18: 233. https://doi.org/10.1186/s12913-018-3054-5](https://doi.org/10.1186/s12913-018-3054-5) | Australia, Brazil, China, the US | Non-fragile (HIC + MIC) | Qualitative method |
| 60 | NCD | [Oyun Chimeddamba, Anna Peeters, Helen L. Walls, and Catherine Joyce. 2015. “Noncommunicable Disease Prevention and Control in Mongolia: A Policy Analysis.” BMC Public Health 15: 660. https://doi.org/10.1186/s12889-015-2040-7](https://doi.org/10.1186/s12889-015-2040-7) | Mongolia | Non-fragile (MIC) | Policy analysis |
| 61 | NCD | Yodi Christiani, Paul Dugdale, Meredith Tavener, and Julie E. Byles. 2016. “The Dynamic of Non-Communicable Disease Control Policy in Indonesia.” *Australian Health Review* 41: 207–213. | Indonesia | Non-fragile (MIC) | Policy analysis |
| 62 | NCD | Rebecca Dodd, Erica Reeve, Emalie Sparks, Anita George, Paula Vivili, Si Thu Win Tin, Dai Buresova, Jacqui Webster, and Anne-Marie Thow. 2020. “The Politics of Food in the Pacific: Coherence and Tension in Regional Policies on Nutrition, the Food Environment and Non-Communicable Diseases.” *Public Health Nutrition* 23 (1): 168–180. doi:10.1017/S1368980019002118 | Twenty-two Pacific island countries and territories | Fragile (MIC) [not for OECD] | Document analysis |
| 63 | NCD | [Beverley M. Essue and Lydia Kapiriri. 2018. “The Unfunded Priorities: An Evaluation of Priority Setting for Noncommunicable Disease Control in Uganda.” Global Health 14: 22. https://doi.org/10.1186/s12992-018-0324-2](https://doi.org/10.1186/s12992-018-0324-2) | Uganda | Fragile (LIC) | Mixed method |
| 64 | NCD | [Karishma S. Furtado, Elizabeth L. Budd, Xiangji Ying, Anna J. deRuyter, Rebecca L. Armstrong, Tahna L. Pettman, Rodrigo S. Reis, Pauline Sung-Chan, Zhaoxin Wang, Tahnee Saunders, Leonardo A. Becker, Jianwei Shi, Long Sum Tabitha Mui, and Ross C. Brownson. 2018. “Exploring Political Influences on Evidence-Based Non-Communicable Disease Prevention Across Four Countries.” Health Education Research 33 (2): 89–103. https://doi.org/10.1093/her/cyy005](https://doi.org/10.1093/her/cyy005) | Australia, Brazil, China and the United States | Non-fragile (HIC + MIC) | Qualitative method |
| 65 | NCD | [Whitney R. Garney, Leigh E. Szucs, Kristin Primm, Laura King Hahn, Kristen M. Garcia, Emily Martin, and Kenneth McLeroy. 2018. “Implementation of Policy, Systems, and Environmental Community-Based Interventions for Cardiovascular Health Through a National Not-for-Profit: A Multiple Case Study.” Health Education and Behavior 45 (6): 855-864. doi:10.1177/1090198118770489](https://doi.org/10.1177/1090198118770489) | USA | Non-fragile (HIC) | Case study |
| 66 | NCD | [Anne C. Grunseit, Samantha Rowbotham, Melanie Crane, Devon Indig, Adrian E. Bauman, and Andrew Wilson. 2019. “Nanny or Canny? Community Perceptions of Government Intervention for Preventive Health.” Critical Public Health 29 (3): 274–289. doi: 10.1080/09581596.2018.1468020](https://doi.org/10.1080/09581596.2018.1468020) | Australian states (New South Wales, Victoria, Tasmania) | Non-fragile (HIC) | Mixed method |
| 67 | NCD | [Pamela A. Juma, Shukri F. Mohamed, Beatrice L. Matanje Mwagomba, Catherine Ndinda, Clarisse Mapa-tassou, Mojisola Oluwasanu, Oladimeji Oladepo, Opeyemi Abiona, Misheck J. Nkhata, Jennifer P. Wisdom, and Jean-Claude Mbanya. 2018. “Non-Communicable Disease Prevention Policy Process in Five African Countries Authors.” BMC Public Health 18: 961. https://doi.org/10.1186/s12889-018-5825-7](https://doi.org/10.1186/s12889-018-5825-7) | Kenya, South Africa, Cameroon, Nigeria, Malawi | Fragile (LIC) + Non-fragile (MIC) | Case study |
| 68 | NCD | [Vivian Lin, Catherine Jones, Shiyong Wang, and Enis Baris. 2014. Health in All Policies as a Strategic Policy Response to NCDs. Health, Nutrition, and Population (HNP) discussion paper. World Bank, Washington, DC. https://openknowledge.worldbank.org/handle/10986/20064](https://openknowledge.worldbank.org/handle/10986/20064) | Global case studies | Global | Document analysis and case study |
| 69 | NCD | [WHO Regional Office for Western Pacific. 2012. Regional Meeting on National Multisectoral Plans for NCD Prevention and Control. Manila : WHO Regional Office for the Western Pacific. http://iris.wpro.who.int/handle/10665.1/12558](http://iris.wpro.who.int/handle/10665.1/12558) | Western Pacific Region of WHO | Non-fragile (HIC + MIC) | Meeting report |
| 70 | NCD | [Johan P. MacKenbach and Martin McKee. 2013. “Social-Democratic Government and Health Policy in Europe: A Quantitative Analysis.” International Journal of Health Services 43 (3): 389–413. doi:10.2190/HS.43.3.b](https://doi.org/10.2190/HS.43.3.b) | Europe | Non-fragile (HIC + MIC) | Quantitative method |
| 71 | NCD | [Roger S. Magnusson and David Patterson. 2014. “The Role of Law and Governance Reform in the Global Response to Non-Communicable Diseases.” Global Health 10: 44. https://doi.org/10.1186/1744-8603-10-44](https://doi.org/10.1186/1744-8603-10-44) | Global | Global | Document analysis |
| 72 | NCD | [Roger S. Magnusson, Benn McGrady, Lawrence Gostin, David Patterson, and Hala Abou Taleb. ‎2019‎. “Legal Capacities Required for Prevention and Control of Noncommunicable Diseases.” Bulletin of the World Health Organization 97 (‎2): 108–117. http://dx.doi.org/10.2471/BLT.18.213777](https://dx.doi.org/10.2471/BLT.18.213777) | Global (examples from India, Uganda, Colombia, USA, Chile, Mexico, SA, UK) | Global [Fragile (LIC) + Non-fragile (HIC/MIC)] | Document analysis |
| 73 | NCD | [Heather Manson, Terrence Sullivan, Phat Ha, Christine Navarro, and José M. Martín-Moreno. 2013. “Goals Are Not Enough: Building Public Sector Capacity for Chronic Disease Prevention.” Public Health Review 35: 11. https://doi.org/10.1007/BF03391696](https://doi.org/10.1007/BF03391696) | Global | Global | Case study |
| 74 | NCD | [Juma, P; Mohamed S & Kyobutungi C. 2017. Analysis of non-communicable disease prevention policies in Kenya. Nairobi: APHRC, http://hdl.handle.net/10625/57553](file:///C:\Users\User\Desktop\Libya%20Best%20Buys\Juma,%20P;%20Mohamed%20S%20&%20Kyobutungi%20C.%202017.%20Analysis%20of%20non-communicable%20disease%20prevention%20policies%20in%20Kenya.%20Nairobi:%20APHRC,%20http:\hdl.handle.net\10625\57553) | Kenya | Non-Fragile (LMIC) | Qualitative method (case study) |
| 75 | NCD | [Shanthi Mendis and Oleg Chestnov. 2013. “Policy Reform to Realize the Commitments of the Political Declaration on Noncommunicable Diseases.” British Medical Bulletin 105 (1): 7–27. https://doi.org/10.1093/bmb/ldt001](https://doi.org/10.1093/bmb/ldt001) | Global | Global | Mixed method |
| 76 | NCD | [Mulenga M. Mukanu, Joseph Mumba Zulu, Chrispin Mweemba, and Wilbroad Mutale. 2017. “Responding to Non-Communicable Diseases in Zambia: A Policy Analysis.” Health Research Policy and Systems 15: 34. https://doi.org/10.1186/s12961-017-0195-7](https://doi.org/10.1186/s12961-017-0195-7) | Zambia | Fragile (LMIC) | Case study |
| 77 | NCD | [Catherine Ndinda, Tidings P. Ndhlovu, Pamela Juma, Gershim Asiki, and Catherine Kyobutungi. 2018. “The Evolution of Non-Communicable Diseases Policies in Post-Apartheid South Africa.” BMC Public Health 18: 956. https://doi.org/10.1186/s12889-018-5832-8 ]](https://doi.org/10.1186/s12889-018-5832-8) | South Affrica | Non-fragile (MIC) | Document analysis |
| 78 | NCD | [Suladda Pongutta, Rapeepong Suphanchaimat, Walaiporn Patcharanarumol, and Viroj Tangcharoensathien. 2019. “Lessons from the Thai Health Promotion Foundation.” Bulletin of the World Health Organization 97 (3): 213–220. https://doi.org/10.2471/BLT.18.220277](https://doi.org/10.2471/BLT.18.220277) | Thailand | Non-fragile (MIC) | Document analysis |
| 79 | NCD | [Linda Richter-Sundberg, Therese Kardakis, Lars Weinehall, Rickard Garvare, and Monica E Nyström. 2015. “Addressing Implementation Challenges During Guideline Development–A Case Study of Swedish National Guidelines for Methods of Preventing Disease.” BMC Health Services Research 15: 19. https://doi.org/10.1186/s12913-014-0672-4](https://doi.org/10.1186/s12913-014-0672-4) | Sweden | Non-fragile (HIC) | Qualitative method (case study) |
| 80 | NCD | [Jianwei Shi, Leiyu Shi, Jinsong Geng, Rui Liu, Xin Gong, Xiaojie Bo, Ning Chen, Qian Liu, Yan Yang, and Zhaoxin Wang. 2019. “Status of Evidence-Based Chronic Diseases Prevention Implementation in Shanghai, China: A Qualitative Study.” International Journal of Health Planning and Management 34 (3): 912–925. https://doi.org/10.1002/hpm.2863](https://doi.org/10.1002/hpm.2863) | China | Non-fragile (UMIC) | Qualitative method |
| 81 | NCD | [Gyambo Sithey, Mu Li, and Anne Marie Thow. 2018. “Strengthening Non-Communicable Disease Policy with Lessons from Bhutan: Linking Gross National Happiness and Health Policy Action.” Journal of Public Health Policy 39: 327–342. https://doi.org/10.1057/s41271-018-0135-y](https://doi.org/10.1057/s41271-018-0135-y) | Bhutan | Non-fragile (LMIC) | Document analysis and interviews |
| 82 | NCD | [Titiporn Tuangratananon, Sangay Wangmo, Nimali Widanapathirana, Suladda Pongutta, Shaheda Viriyathorn, Walaiporn Patcharanarumol, Kouland Thin, Somil Nagpal, Christian Edward L. Nuevo, Retna Siwi Padmawati, Maria Elizabeth Puyat-Murga, Laksono Trisnantoro, Kinzang Wangmo, Nalinda Wellappuli, Phuong Hoang Thi, Tuan Khuong Anh, Thinley Zangmo, and Viroj Tangcharoensathien. 2019. “Implementation of National Action Plans on Noncommunicable Diseases, Bhutan, Cambodia, Indonesia, Philippines, Sri Lanka, Thailand and Viet Nam.” Bulletin of the World Health Organization 97 (2): 129–141. https://doi.org/10.2471/BLT.18.220483](https://doi.org/10.2471/BLT.18.220483) | Bhutan, Cambodia, Indonesia, Philippines, Sri Lanka, Thailand and Viet Nam. | Non-fragile (MIC) | Document analysis and interviews |
| 83 | NCD | [Nigel Unwin, T. Alafia Samuels, Trevor Hassell, Ross C. Brownson, and Cornelia Guell. 2017. “The Development of Public Policies to Address Non-Communicable Diseases in the Caribbean Country of Barbados: The Importance of Problem Framing and Policy Entrepreneurs.” International Journal of Health Policy and Management 6 (2): 71–82. https://doi.org/10.15171/ijhpm.2016.74](https://doi.org/10.15171/ijhpm.2016.74) | Barbados | Non-fragile (HIC) | Mixed method |
| 84 | NCD | [Baktygul Akkazieva, Juan Tello, Barton Smith, Melitta Jakab, Konstantin Krasovsky, Nina Sautenkova, Lola Yuldasheva, and Mekhre Shoismatyuloeva‎. 2015. Better Non-Communicable Disease Outcomes: Challenges and Opportunities for Health Systems. Tajikistan Country Assessment. Regional Office for Europe, World Health Organization, Copenhagen. https://apps.who.int/iris/handle/10665/153907](https://apps.who.int/iris/handle/10665/153907) | Tajikistan | Non-fragile (LIC) | Case study |
| 85 | NCD | [Yong Ho Khang. 2013. “Burden of Noncommunicable Diseases and National Strategies to Control Them in Korea.” Journal of Preventive Medicine and Public Health 46 (4): 155–164. https://doi.org/10.3961/jpmph.2013.46.4.155](https://doi.org/10.3961/jpmph.2013.46.4.155) | South Korea | Non-fragile (LIC) | Case study |
| 86 | NCD | [Regional Office for the Western Pacific. 2017. Progress on the Prevention and Control of Noncommunicable Diseases in the Western Pacific Region: Country Capacity Survey. Regional Office for the Western Pacific, World Health Organization, Manila. http://iris.wpro.who.int/handle/10665.1/14162](http://iris.wpro.who.int/handle/10665.1/14162) | Western Pacific region | Fragile + Non-fragile (HIC + MIC) | Survey |
| 87 | NCD, medicines | [Jordan D. Jarvis, Hannah Woods , Anjli Bali , Efosa Oronsaye, and Nav Persaud. 2019. “Selection of WHO-Recommended Essential Medicines for Non-Communicable Diseases on National Essential Medicines Lists.” PLoS ONE 14 (8): e0220781. https://doi.org/10.1371/journal.pone.0220781](https://doi.org/10.1371/journal.pone.0220781) | China | Non-fragile (UMIC) | Qualitative method |
| 88 | Obesity | [Lucy C. Farrell, Vivienne M. Moore, Megan J. Warin, and Jackie M. Street. 2019. “Why Do the Public Support or Oppose Obesity Prevention Regulations? Results from a South Australian Population Survey.” Health Promotion Journal of Australia 30 (1): 47–59. https://doi.org/10.1002/hpja.185](https://doi.org/10.1002/hpja.185) | Bhutan | Non-fragile (LMIC) | Document analysis and interviews |
| 89 | Diet | [Mohammad Amerzadeh and Amirhossein Takian. 2020. “Reducing Sugar, Fat, and Salt for Prevention and Control of Noncommunicable Diseases (NCDs) as an Adopted Health Policy in Iran.” Medical Journal of the Islamic Republic of Iran 34: 136. https://doi.org/10.34171/mjiri.34.136](https://doi.org/10.34171/mjiri.34.136) | Iran | Fragile (UMIC) | Mixed method |
| 90 | Physical activity | [Rodney S. Lyn, Erica R. Sheldon, and Michael P. Eriksen. 2017. “Adopting State-Level Policy to Support Physical Activity Among School-Aged Children and Adolescents: Georgia’s SHAPE Act.” Public Health Reports 132 (2_suppl): 9S–15S. doi:10.1177/0033354917719705](https://doi.org/10.1177/0033354917719705) | Australia | Non-fragile (HIC) | Survey |
| 91 | Physical activity | [Madhuvanti M. Murphy, Nigel Unwin, T. Alafia Samuels, Trevor A Hassell, Lisa Bishop, and Cornelia Guell. 2018. “Evaluating Policy Responses to Noncommunicable Diseases in Seven Caribbean Countries: Challenges to Addressing Unhealthy Diets and Physical Inactivity.” Pan American Journal of Public Health 42: e174. https://doi.org/10.26633/RPSP.2018.174](https://doi.org/10.26633/RPSP.2018.174) | Caribbean region | Non-fragile (HIC + MIC) | Qualitative method |
| 92 | Physical activity | [Christopher E. Politis, David L. Mowat, and Deb Keen. 2017. “Pathways to Policy: Lessons Learned in Multisectoral Collaboration for Physical Activity and Built Environment Policy Development from the Coalitions Linking Action and Science for Prevention (CLASP) Initiative.” Canadian Journal of Public Health 108 (2): e192–e198. https://doi.org/10.17269/cjph.108.5758](https://doi.org/10.17269/cjph.108.5758) | Canada | Non-fragile (HIC) | Qualitative method |
| 93 | Physical activity | [Katie A. Weatherson, Rhyann McKay, Heather L. Gainforth, and Mary E. Jung. 2017. “Barriers and Facilitators to the Implementation of a School-Based Physical Activity Policy in Canada: Application of the Theoretical Domains Framework.” BMC Public Health 17: 83. https://doi.org/10.1186/s12889-017-4846-y](https://doi.org/10.1186/s12889-017-4846-y) | Canada | Non-fragile (HIC) | Qualitative method |
| 94 | Physical activity, diet | [Saskia Muellmann, Berit Steenbock, Katrien De Cocker, Marieke De Craemer, Catherine Hayes, Miriam P. O’Shea, Karolina Horodyska, Justyna Bell, Aleksandra Luszczynska, Gun Roos, Lars Jørun Langøien, Gro Rugseth, Laura Terragni, Ilse De Bourdeaudhuij, Johannes Brug, and Claudia R. Pischke. 2017. “Views of Policy Makers and Health Promotion Professionals on Factors Facilitating Implementation and Maintenance of Interventions and Policies Promoting Physical Activity and Healthy Eating: Results of the DEDIPAC Project.” BMC Public Health 17: 932. https://doi.org/10.1186/s12889-017-4929-9](https://doi.org/10.1186/s12889-017-4929-9) | Europe | Non-fragile (HIC) | Mixed method |
| 95 | Physical activity, diet | [Mojisola Oluwasanu, Opeyemi Oladunni, amd Oladimeji Oladepo. 2020. “Multisectoral Approach and WHO ‘Bestbuys’ in Nigeria’s Nutrition and Physical Activity Policies.” Health Promotion International 35 (6): 1383–1393. https://doi.org/10.1093/heapro/daaa009](https://doi.org/10.1093/heapro/daaa009) | Nigeria | Fragile (LMIC) | Case study |
| 96 | Physical activity | Fiona Bull, Karen Milton, Sonja Kahlmeier, Alberto Arlotti, Andrea Backović Juričan, Olov Belander, Brian Martin, Eva Martin-Diener, Ana Marques, Jorge Mota, Tommi Vasankari, and Anita Vlasveld. 2015. “Turning the Tide: National Policy Approaches to Increasing Physical Activity in Seven European Countries.” *British Journal of Sports Medicine* 49: 749–56. | Finland, Italy, the Netherlands, Norway, Portugal, Slovenia and Switzerland | Non-fragile (HIC) | Policy analysis |
| 97 | Salt | [Adriana Blanco-Metzler, María de los Ángeles Montero-Campos, Hilda Núñez-Rivas, Cecilia Gamboa-Cerda, and Germana Sánchez. 2012. “Avances en la reducción del consumo de sal y sodio en Costa Rica [Advances in Reducing Salt and Sodium Intake in Costa Rica].” Revista Panamericana de Salud Publica 32 (4): 316–320. https://doi.org/10.1590/s1020-49892012001000011](https://doi.org/10.1590/s1020-49892012001000011) | Costa Rica | Non-fragile (UMIC) | Document analysis |
| 98 | Salt | [Norm Campbell, Barbara Legowski, Branka Legetic, Daniel Ferrante, Eduardo Nilson, Christine Campbell, and Mary L'Abbé. 2014. “Targets and Timelines for Reducing Salt in Processed Food in the Americas.” Journal of Clinical Hypertension 16 (9): 619–623. https://doi.org/10.1111/jch.12379](https://doi.org/10.1111/jch.12379) | 8 countries in the Americas region | Non-fragile (HIC + MIC) | Policy analysis |
| 99 | Salt | [Luciana Castronuovo, Lorena Allemandi, Victoria Tiscornia, Beatriz Champagne, Norm Campbell, and Verónica Schoj. 2017. “Analysis of a Voluntary Initiative to Reduce Sodium in Processed and Ultra-Processed Food Products in Argentina: The Views of Public and Private Sector Representatives.” Cadernos de Saude Publica 33 (6): e00014316. https://doi.org/10.1590/0102-311X00014316](https://doi.org/10.1590/0102-311X00014316) | Argentina | Non-fragile (UMIC) | Qualitative method |
| 100 | Salt | [Priti Gupta, Sailesh Mohan, Claire Johnson, Vandana Garg, Sudhir Raj Thout, Roopa Shivashankar, Anand Krishnan, Bruce Neal, and Dorairaj Prabhakaran. 2018. “Stakeholders’ Perceptions Regarding a Salt Reduction Strategy for India: Findings from Qualitative Research.” PLoS ONE 13 (8): e0201707. https://doi.org/10.1371/journal.pone.0201707](https://doi.org/10.1371/journal.pone.0201707) | India | Non-fragile (MIC) | Qualitative method |
| 101 | Salt | [F. He, H. Brinsden, and G. MacGregor. 2014. “Salt Reduction in the United Kingdom: A Successful Experiment in Public Health.” Journal of Human Hypertension 28: 345–352. https://doi.org/10.1038/jhh.2013.105](https://doi.org/10.1038/jhh.2013.105) | UK | Non-fragile (HIC) | Policy analysis |
| 102 | Salt | [Branka Legetic and Norm Campbell. 2011. “Reducing Salt Intake in the Americas: Pan American Health Organization Actions.” Journal of Health Communication 16 Suppl 2: 37–48. https://doi.org/10.1080/10810730.2011.601227](https://doi.org/10.1080/10810730.2011.601227) | America region | Non-fragile (HIC + MIC) | Case study |
| 103 | Salt | Shahram Rafieifar, Hamed Pouraram, Abolghassem Djazayery, Fereydoun Siassi, Zahra Abdollahi, Ahmad Reza Dorosty, Mitra Abtahi, Hossein Kazemeini, and Farshad Farzadfar. 2016. “Strategies and Opportunities Ahead to Reduce Salt Intake.” *Archives of Iranian Medicine* 19 (10): 729–734. | Iran | Fragile (UMIC) | Document analysis |
| 104 | Salt | Belinda Reeve and Roger Magnusson. 2015. “Food Reformulation and the (Neo)-Liberal State: New Strategies for Strengthening Voluntary Salt Reduction Programs in the UK and USA.” *Public Health* 129 (8): 1061–1073. | UK, USA | Non-fragile (HIC) | Document analysis |
| 105 | Salt | [Kathy Trieu, Jacqui Webster, Stephen Jan, Silvia Hope, Take Naseri, Merina Ieremia, Colin Bell, Wendy Snowdon, and Marj Moodie. 2018. “Process Evaluation of Samoa’s National Salt Reduction Strategy (MASIMA): What Interventions Can Be Successfully Replicated in Lower-Income Countries?” Implementation Science 13: 107. https://doi.org/10.1186/s13012-018-0802-1](https://doi.org/10.1186/s13012-018-0802-1) | Samoa | Non-fragile (UMIC) | Process evaluation and surveys |
| 106 | Diet | [Amos Laar, Amy Barnes, Richmond Aryeetey, Akua Tandoh, Kristin Bash, Kobby Mensah, Francis Zotor, Stefanie Vandevijvere, and Michelle Holdsworth. 2020. “Implementation of Healthy Food Environment Policies to Prevent Nutrition-Related Non-Communicable Diseases in Ghana: National Experts' Assessment of Government Action.” Food Policy 93, 101907. https://doi.org/10.1016/j.foodpol.2020.101907](https://doi.org/10.1016/j.foodpol.2020.101907) | Ghana | Non-fragile (LMIC) | Policy analysis |
| 107 | Salt | [Karen Charlton, Jacqui Webster, and Paul Kowal. 2014. “To Legislate or Not to Legislate? A Comparison of the UK and South African Approaches to the Development and Implementation of Salt Reduction Programs.” Nutrients 6 (9): 3672–3695. https://doi.org/10.3390/nu6093672](https://doi.org/10.3390/nu6093672) | UK and South Africa | Non-fragile (HIC + MIC) | Case study |
| 108 | Sugar | [Carlos M. Guerrero-López, Mishel Unar-Munguía, and M. Arantxa Colchero. 2017. “Price Elasticity of the Demand for Soft Drinks, Other Sugar-Sweetened Beverages and Energy Dense Food in Chile.” BMC Public Health 17: 180. https://doi.org/10.1186/s12889-017-4098-x](https://doi.org/10.1186/s12889-017-4098-x) | Mexico | Non-fragile (UMIC) | Quantitative method |
| 109 | Sugar | [Susan Greenhalgh. 2019. “Soda Industry Influence on Obesity Science and Policy in China.” Journal of Public Health Policy 40: 5–16. https://doi.org/10.1057/s41271-018-00158-x](https://doi.org/10.1057/s41271-018-00158-x) | China | Non-fragile (UMIC) | Qualitative method |
| 110 | Sugar | [Alex Myers, David Fig, Aviva Tugendhaft, Jonathan E. Myers, and Karen J. Hofman 2017. “The History of the South African Sugar Industry Illuminates Deeply Rooted Obstacles for Sugar Reduction Anti-Obesity Interventions.” African Studies 76 (4): 475–490. doi: 10.1080/00020184.2017.1311515](https://doi.org/10.1080/00020184.2017.1311515) | South Africa | Non-fragile (UMIC) | Case study |
| 111 | Sugar | [Frances Onagan, Beverly Ho, and Karl Kendrick Chua. 2019. “Development of a Sweetened Beverage Tax, Philippines.” Bulletin of the World Health Organization 97 (2): 154–159. https://doi.org/10.2471/BLT.18.220459](https://doi.org/10.2471/BLT.18.220459) | Philippines | Non-fragile (UMIC) | Document analysis |
| 112 | Sugar | Vicente Ortún, Beatriz G López-Valcárcel, and Jaime Pinilla. 2016. “Tax on Sugar Sweetened Beverages in Spain.” *Revista Española de Salud Pública* 13 (90) :e1-e13. | Multi-country (Spain, Mexico) | Non-fragile (HIC + MIC) | Document analysis |
| 113 | Sugar | [Christina A. Roberto and Jennifer L. Pomeranz. 2015. “Public Health and Legal Arguments in Favor of a Policy to Cap the Portion Sizes of Sugar-Sweetened Beverages.” American Journal of Public Health 105 (11): 2183–2190. https://doi.org/10.2105/AJPH.2015.302862](https://doi.org/10.2105/AJPH.2015.302862) | New York, US | Non-fragile (HIC) | Document analysis |
| 114 | Sugar | [Alex Myers, David Fig, Aviva Tugendhaft, Jessie Mandle, Jonathan Myers, and Karen Hofman. 2017. “Sugar and Health in South Africa: Potential Challenges to Leveraging Policy Change.” Global Public Health 12 (1): 98–115. doi: 10.1080/17441692.2015.1071419](https://doi.org/10.1080/17441692.2015.1071419) | South Africa | Non-fragile (UMIC) | Case study |
| 115 | Tobacco | [Naowarut Charoenca, Jeremiah Mock, Nipapun Kungskulniti, Sunida Preechawong, Nicholas Kojetin, and Stephen L. Hamann. 2012. “Success Counteracting Tobacco Company Interference in Thailand: An Example of FCTC Implementation for Low- and Middle-income Countries.” International Journal of Environmental Researchand Public Health 9: 1111–1134. https://doi.org/10.3390/ijerph9041111](https://doi.org/10.3390/ijerph9041111) | Thailand | Non-fragile (MIC) | Qualitative method |
| 116 | Tobacco | [Jeff Collin, Sarah E. Hill, Mor Kandlik Eltanani, Evgeniya Plotnikova, Rob Ralston, and Katherine E. Smith. 2017. “Can Public Health Reconcile Profits and Pandemics? An Analysis of Attitudes to Commercial Sector Engagement in Health Policy and Research.” PLoS ONE 12 (9): e0182612. https://doi.org/10.1371/journal.pone.0182612](https://doi.org/10.1371/journal.pone.0182612) | 40 countries (high, middle and low income) | Global | Survey |
| 117 | Tobacco | [Eric Crosbie, George Thomson, Becky Freeman, and Stella Bialous. 2018. “Advancing Progressive Health Policy to Reduce NCDs Amidst International Commercial Opposition: Tobacco Standardised Packaging in Australia.” Global Public Health 13 (12): 1753–1766. doi: 10.1080/17441692.2018.1443485](https://doi.org/10.1080/17441692.2018.1443485) | Australia | Non-fragile (HIC) | Mixed method |
| 118 | Tobacco | [Vera Luiza da Costa e Silva, Daniela Pantani, Mônica Andreis, Robert Sparks, and Ilana Pinsky. 2013. “Bridging the Gap Between Science and Public Health: Taking Advantage of Tobacco Control Experience in Brazil to Inform Policies to Counter Risk Factors for Non-Communicable Diseases.” Addiction 108 (8): 1360–1366. https://doi.org/10.1111/add.12203](https://doi.org/10.1111/add.12203) | Brazil | Non-fragile (UMIC) | Case study |
| 119 | Tobacco | [Amanda Fallin, Amie Goodin, Mary Kay Rayens, Sarah Morris, and Ellen J. Hahn. 2014. “Smoke-Free Policy Implementation: Theoretical and Practical Considerations.” Policy, Politics, and Nursing Practice 15 (3-4): 81–92. doi:10.1177/1527154414562301](https://doi.org/10.1177/1527154414562301) | USA | Non fragile (HIC) | Document analysis |
| 120 | Tobacco | [Soumita Ghose, Alok Sardar, Suman Shiva, Brega Ellen Mullan, and Soumitra S Datta. 2019. “Perception of Tobacco Use in Young Adults in Urban India: A Qualitative Exploration with Relevant Health Policy Analysis.” Ecancermedicalscience 13: 915. https://doi.org/10.3332/ecancer.2019.915](https://doi.org/10.3332/ecancer.2019.915) | India | Non-fragile (LMIC) | Qualitative method |
| 121 | Tobacco | [Holly Jarman. 2013. “Attack on Australia: Tobacco Industry Challenges to Plain Packaging.” Journal of Public Health Policy 34: 375–387. https://doi.org/10.1057/jphp.2013.18](https://doi.org/10.1057/jphp.2013.18) | Australia | Non-fragile (HIC) | Document analysis |
| 122 | Tobacco | [Ronald Labonté, Raphael Lencucha, Fastone Goma, Richard Zulu, and Jeffrey Drope. 2019. “Consequences of Policy Incoherence: How Zambia's Post-FCTC Investment Policy Stimulated Tobacco Production.” Journal of Public Health Policy 40 (3): 286–291. https://doi.org/10.1057/s41271-019-00171-8](https://doi.org/10.1057/s41271-019-00171-8) | Zambia | Fragile (LMIC) | Qualitative method |
| 123 | Tobacco | [Jennifer Leeman, Allison Myers, Jennifer C. Grant, Mary Wangen, and Tara L. Queen. 2017. “Implementation Strategies to Promote Community-Engaged Efforts to Counter Tobacco Marketing at the Point of Sale.” Translational Behavioral Medicine 7 (3): 405–414. https://doi.org/10.1007/s13142-017-0489-x](https://doi.org/10.1007/s13142-017-0489-x) | USA | Non-fragile (HIC) | Qualitative method |
| 124 | Tobacco | [Raphael Lencucha, Jeffrey Drope, and Jenina Joy Chavez. 2015. “Whole-of-Government Approaches to NCDs: The Case of the Philippines Interagency Committee-Tobacco.” Health Policy and Planning 30 (7): 844–852. https://doi.org/10.1093/heapol/czu085](https://doi.org/10.1093/heapol/czu085) | Philippines | Non-fragile (MIC) | Mixed method |
| 125 | Tobacco | [Clarisse Mapa-Tassou, Cecile Rénée Bonono, Felix Assah, Jennifer Wisdom, Pamela A. Juma, Jean-Claude Katte, Zakariaou Njoumemi, Pierre Ongolo-Zogo, Leopold K. Fezeu, Eugene Sobngwi, and Jean Claude Mbanya. 2018. “Two Decades of Tobacco Use Prevention and Control Policies in Cameroon: Results from the Analysis of Non-Communicable Disease Prevention Policies in Africa.” BMC Public Health 18: 958 2018. https://doi.org/10.1186/s12889-018-5828-4](https://doi.org/10.1186/s12889-018-5828-4) | Cameroon | Fragile (LMIC) | Case study |
| 126 | Tobacco | [Lazarous Mbulo, Nwokocha Ogbonn, Isiaka Olarewaju, Emmanuel Musa, Simone Salandy, Nivo Ramanandraibe, and Krishna Palipudi on behalf of GATS collaborative group. 2016. “Preventing Tobacco Epidemic in LMICs with Low Tobacco Use—Using Nigeria GATS to review WHO MPOWER Tobacco Indicators and Prevention Strategies.” Preventive Medicine 91S: S9–S15. https://doi.org/10.1016/j.ypmed.2016.04.005](https://doi.org/10.1016/j.ypmed.2016.04.005) | Nigeria | Fragile (LMIC) | Survey |
| 127 | Tobacco | [Ashleigh Cussen and Judith McCool. 2011. “Tobacco Promotion in the Pacific: The Current State of Tobacco Promotion Bans and Options for Accelerating Progress.” Asia Pacific Journal of Public Health 23 (1): 70–78. doi:10.1177/1010539510390925](https://doi.org/10.1177/1010539510390925) | Western Pacific Region (WPR) | Fragile (MIC) [not for OECD] | Document analysis |
| 128 | Tobacco | [Judith McCool, Jeanie McKenzie, Annabel Lyman, and Matthew Allen. 2013. “Supporting Pacific Island Countries to Strengthen Their Resistance to Tobacco Industry Interference in Tobacco Control: A Case Study of Papua New Guinea and Solomon Islands.” International Journal of Environmental Research and Public Health 10 (8): 3424–3434. https://doi.org/10.3390/ijerph10083424](https://doi.org/10.3390/ijerph10083424) | Papua New Guinea and Solomon Islands | Fragile (MIC) | Document analysis |
| 129 | Tobacco | [Shukri F. Mohamed, Pamela Juma, Gershim Asiki, and Catherine Kyobutungi. 2018. “Facilitators and Barriers in the Formulation and Implementation of Tobacco Control Policies in Kenya: A Qualitative Study.” BMC Public Health 18: 960. https://doi.org/10.1186/s12889-018-5830-x](https://doi.org/10.1186/s12889-018-5830-x) | Kenya | Fragile (LMIC) | Case study |
| 130 | Tobacco | [Oladimeji Oladepo, Mojisola Oluwasanu, and Opeyemi Abiona. 2018. “Analysis of Tobacco Control Policies in Nigeria: Historical Development and Application of Multi-Sectoral Action.” BMC Public Health 18: 959 2018. https://doi.org/10.1186/s12889-018-5831-9](https://doi.org/10.1186/s12889-018-5831-9) | Nigeria | Fragile (LMIC) | Case study |
| 131 | Tobacco | [Hilal Ozcebe, Toker Erguder, Mehmet Balcilar, Pavel Ursu, Aaron Reeves, David Stuckler, Andrew Snell, Gauden Galea, Bente Mikkelsen, and Kristina Mauer-Stender. 2018. “The Perspectives of Politicians on Tobacco Control in Turkey.” European Journal of Public Health 28 (suppl_2): 17–21. https://doi.org/10.1093/eurpub/cky152](https://doi.org/10.1093/eurpub/cky152) | Turkey | Non-fragile (UMIC) | Mixed method |
| 132 | Tobacco | Jalal Poorolajal, Younes Mohammadi, and Azam Mahmoodi. 2017. “Challenges of Tobacco Control Program in Iran.” *Archives of Iranian Medicine* 20 (4): 229–234. | Iran | Fragile (UMIC) | Survey |
| 133 | Tobacco | [Erica Cavalcanti Rangel, Andre Pereira Neto, Tania Maria Cavalcante, Egléubia Andrade Oliveira, and Vera Luiza da Costa e Silva. 2017. “The Decision-Making Process in Brazil's Ratification of the World Health Organization Framework Convention on Tobacco Control.” Cadernos de Saude Publica 33 (Suppl 3): e00126115. https://doi.org/10.1590/0102-311X00126115](https://doi.org/10.1590/0102-311X00126115) | Brasil | Non-fragile (UMIC) | Qualitative method |
| 134 | Tobacco | [Lindsay Robertson, Louise Marsh, Janet Hoek, Rob McGee, and Richard Egan. 2015. “Regulating the Sale of Tobacco in New Zealand: A Qualitative Analysis of Retailers' Views and Implications for Advocacy.” International Journal on Drug Policy 26 (12): 1222–1230. https://doi.org/10.1016/j.drugpo.2015.08.015](https://doi.org/10.1016/j.drugpo.2015.08.015) | New Zealand | Non-fragile (HIC) | Qualitative method |
| 135 | Tobacco | Michal Stoklosa and Hana Ross. 2014. “Tobacco Control Funding for Low-Income and Middle-Income Countries in a Time of Economic Hardship.” *Tobacco Control* 23: e122–e126. | LMICs | Global | Case study |
| 136 | Tobacco | [Saliyou Sanni, Charles Hongoro, Catherine Ndinda, and Jennifer P. Wisdom. 2018. “Assessment of the Multi-Sectoral Approach to Tobacco Control Policies in South Africa and Togo.” BMC Public Health 18: 962. https://doi.org/10.1186/s12889-018-5829-3](https://doi.org/10.1186/s12889-018-5829-3) | South Africa and Togo | Fragile (LIC) + Non-fragile (HIC/MIC) | Document analysis |
| 137 | Tobacco | [Travis D. Satterlund, Diana Cassady, Jeanette Treiber, and Cathy Lemp. 2011. “Barriers to Adopting and Implementing Local-Level Tobacco Control Policies.” Journal of Community Health 36 (4): 616–623. https://doi.org/10.1007/s10900-010-9350-6](https://doi.org/10.1007/s10900-010-9350-6) | California, USA | Non-fragile (HIC) | Document analysis |
| 138 | Tobacco | [World Health Organization and WHO Framework Convention on Tobacco Control. ‎2020‎. WHO FCTC Implementation Review in Pacific Island Countries. Geneva: World Health Organization. https://apps.who.int/iris/handle/10665/337194.](https://apps.who.int/iris/handle/10665/337194) | Pacific island countries | Fragile (MIC) [not for OECD] | Case study |
| 139 | Tobacco | [Jennifer P. Wisdom, Pamela Juma, Beatrice Mwagomba, Catherine Ndinda, Clarisse Mapa-Tassou, Felix Assah, Misheck Nkhata, Shukri F. Mohamed, Oladepo Oladimeji, Opeyemi Oladunni, Mojisola Oluwasanu, Saliyou Sanni, Jean-Claude Mbanya, and Catherine Kyobutungi. 2018. “Influence of the WHO Framework Convention on Tobacco Control on Tobacco Legislation and Policies in Sub-Saharan Africa.” BMC Public Health 18: 954 2018. https://doi.org/10.1186/s12889-018-5827-5](https://doi.org/10.1186/s12889-018-5827-5) | Sub-Saharan Africa (Cameroon, Kenya, Nigeria, Malawi, South Africa, and Togo) | Fragile (LIC) + Non-fragile (MIC) | Case study |
| 140 | Tobacco | World Bank Group. 2018. Reducing Tobacco Use Through Taxation: The Experience of the Republic of Korea. World Bank, Washington, DC. https://openknowledge.worldbank.org/handle/10986/30020 | Republic of Korea | Non fragile (HIC) | Case study |
| 141 | Tobacco | World Bank Group. 2018. Advancing Action on the Implementation of Tobacco Tax Harmonization in the Organization of Eastern Caribbean States Countries. World Bank, Washington, DC. https://openknowledge.worldbank.org/handle/10986/30034 | Carribean Island countries | Non-fragile (MIC) | Case study |
| 142 | Tobacco | Robert Beaglehole, Ruth Bonita, Derek Yach, Judith Mackay, and K. Srinath Reddy. 2015. “A Tobacco-Free World: A Call to Action to Phase Out the Sale of Tobacco Products by 2040.” *Lancet* 385 (9972): 1011–1018. | Global | Global | Case study |
| 143 | Tobacco | Anna B. Gilmore, Gary Fooks, Jeffrey Drope, Stella Aguinaga Bialous, and Rachel Rose Jackson. 2015. “Exposing and Addressing Tobacco Industry Conduct in Low-Income and Middle-Income Countries.” *Lancet* 385 (9972): 1029–1043. | LMICs | Non-fragile (LMIC) | Case study |
| 144 | Tobacco | Judith Mackay, Bungon Ritthiphakdee, and K. Srinath Reddy. 2013. “Tobacco Control in Asia.” *Lancet* 381 (9877): 1581–1587. | WHO’s combined South-East Asia and Western Pacific regions | Non-fragile (HIC+MiC) | Document analysis |
| 145 | Tobacco | Gonghuan Yang, Yu Wang, Yiqun Wu, Jie Yang, and Xia Wan. 2015. “The Road to Effective Tobacco Control in China.” *Lancet* 385 (9972): 1019–1028. | China | Non-fragile (UMIC) | Case study |
| 146 | Tobacco, food, alcohol | [Raphael Lencucha and Anne Marie Thow. 2019. “How Neoliberalism Is Shaping the Supply of Unhealthy Commodities and What This Means for NCD Prevention.” International Journal of Health Policy and Management 8 (9): 514–520. https://doi.org/10.15171/ijhpm.2019.56](https://doi.org/10.15171/ijhpm.2019.56) | Examples from Malawi, Zambia, Philippines | Fragile + Non-fragile | Prospective analysis |
| 147 | Trans fatty acids | [Andrey Demin, Bianca Løge, Olga Zhiteneva, Chizuru Nishida, Stephen Whiting, Holly Rippin, Christian Delles, Saliya Karymbaeva, Kremlin Wickramasinghe, and João Breda. 2020. “Trans Fatty Acid Elimination Policy in Member States of the Eurasian Economic Union: Implementation Challenges and Capacity for Enforcement.” Journal of Clinical Hypertension 22 (8): 1328–1337. https://doi.org/10.1111/jch.13945](https://doi.org/10.1111/jch.13945) | Europe | Non fragile (HIC) | Case study |
| 148 | Diet | [Sareh Edalati, Nasrin Omidvar, Arezoo Haghighian Roudsari, Delaram Ghodsi, and Azizollaah Zargaraan. 2020. “Development and Implementation of Nutrition Labelling in Iran: A Retrospective Policy Analysis.” International Journal of Health Planning and Management 35 (1): e28–e44. https://doi.org/10.1002/hpm.2924](https://doi.org/10.1002/hpm.2924) | Iran | Fragile (MIC) | Policy analysis |
| 149 | Diet | [Yuvaraj Krishnamoorthy, Karthika Ganesh, and Manikandanesan Sakthivel. 2020. “Fat Taxation in India: A Critical Appraisal of Need, Public Health Impact, and Challenges in Nationwide Implementation.” Health Promotion Perspectives 10 (1): 8–12. https://doi.org/10.15171/hpp.2020.04](https://doi.org/10.15171/hpp.2020.04) | India | Non fragile (MIC) | Case study |
| 150 | Primary care | [Lwin Lwin Aye, Jaya Prasad Tripathy, Thae Maung Maung, Myo Minn Oo, Mya Lay Nwe, Hlaing Moh Moh Thu, Ko Ko, and Kyaw Kan Kaung. 2020. “Experiences from the Pilot Implementation of the Package of Essential Non-Communicable Disease Interventions (PEN) in Myanmar, 2017-18: A Mixed Methods Study.” PLoS ONE 15 (2): e0229081. https://doi.org/10.1371/journal.pone.0229081](https://doi.org/10.1371/journal.pone.0229081) | Myanmar | Fragile (MIC) | Mixed method |
| 151 | NCD | Ankita Meghani, Charles Ssemugabo, George Pariyo, Adnan A. Hyder, Elizeus Rutebemberwa, and Dustin G. Gibson. 2021. “Curbing the Rise of Noncommunicable Diseases in Uganda: Perspectives of Policy Actors.” *Global Health, Science and Practice* 9 (1): 149-159. doi: 10.9745/GHSP-D-20-00051 | Uganda | Fragile (LIC) | Qualitative method |
| 152 | NCD | [Taha Nasiri, Shahram Yazdani, Lida Shams, and Amirhossein Takian. 2021. "Stewardship of Noncommunicable Diseases in Iran: A Qualitative Study." International Journal of Health Governance 26 (2): 179–198. https://doi.org/10.1108/IJHG-07-2020-0074](https://doi.org/10.1108/IJHG-07-2020-0074) | Iran | Fragile (MIC) | Qualitative method |
| 153 | CVD | [Rawlance Ndejjo, Rhoda K. Wanyenze, Fred Nuwaha, Hilde Bastiaens, and Geofrey Musinguzi. 2020. “Barriers and Facilitators of Implementation of a Community Cardiovascular Disease Prevention Programme in Mukono and Buikwe Districts in Uganda Using the Consolidated Framework for Implementation Research.” Implementation Science 15: 106. https://doi.org/10.1186/s13012-020-01065-0](https://doi.org/10.1186/s13012-020-01065-0) | Uganda | Fragile (LIC) | Qualitative method |
| 154 | NCD | [L.O. Gostin, H. Abou-Taleb, S.A. Roache, and A. Alwan. 2017. “Legal Priorities for Prevention of non-Communicable Diseases: Innovations from WHO's Eastern Mediterranean Region.” Public Health 144: 4–12. https://doi.org/10.1016/j.puhe.2016.11.001](https://doi.org/10.1016/j.puhe.2016.11.001) | Easter Mediterranean Region | Fragile + Non-fragile | Document analysis |
| 155 | Cancer | P.J. Maver and M. Poljak. 2020. “Primary HPV-Based Cervical Cancer Screening in Europe: Implementation Status, Challenges, and Future Plans.” *Clinical Microbiology and Infection* 26 (5): 579–583. | Europe | Non fragile (HIC) | Mixed method |
| 156 | Cancer, HPV screening | [Megan Bernstein, Anjali Hari, Sahil Aggarwal, Debora Lee, Allison Farfel, Priya Patel, Kaavya Raman, Shella Raja, Reece Fenning, Mark Lieber, William Minteer, Sean Denny, and Maureen Ries. 2018. “Implementation of a Human Papillomavirus Screen-and-Treat Model in Mwanza, Tanzania: Training Local Healthcare Workers for Sustainable Impact.” International Health 10 (3): 197–201. https://doi.org/10.1093/inthealth/ihy014](https://doi.org/10.1093/inthealth/ihy014) | Tanzania | Non-fragile (MIC) | Mixed method |
| 157 | Diet | [Marco Zenone, Diego Silva, Julia Smith, and Kelley Lee. 2021. “How Does the British Soft Drink Association Respond to Media Research Reporting on the Health Consequences of Sugary Drinks?” Global Health 17: 72. https://doi.org/10.1186/s12992-021-00719-y](https://doi.org/10.1186/s12992-021-00719-y) | UK | Non fragile (HIC) | Document analysis |
| 158 | Diet | [Anne Marie Thow, Safura Abdool Karim, Mulenga M Mukanu, Gemma Ahaibwe, Milka Wanjohi, Lebogang Gaogane, Hans Justus Amukugo, Charles Mulindabigwi Ruhara, Twalib Ngoma, Gershim Asiki, Agnes Erzse, and Karen Hofman. 2021. “The Political Economy of Sugar-Sweetened Beverage Taxation: An Analysis from Seven Countries In Sub-Saharan Africa.” Global Health Action 14 (1): 1909267. https://doi.org/10.1080/16549716.2021.1909267](https://doi.org/10.1080/16549716.2021.1909267) | 7 countries in Sub-Saharan Africa | Fragile + Non-fragile | Document analysis |
| 159 | Tobacco | [Owuraku Kusi-Ampofo. 2021. “Negotiating Change: Ideas, Institutions, and Political Actors in Tobacco Control Policy Making in Mauritius.” Journal of Health Politics and Policy Law 46 (3): 435–465. doi: https://doi.org/10.1215/03616878-8893543](https://doi.org/10.1215/03616878-8893543) | Mauritius | Non-fragile (MIC) | Case study |
| 160 | Tobacco | [Echezona Ejike Udokanma, Ikedinachi Ogamba, and Cajetan Ilo. 2021. “A Health Policy Analysis of the Implementation of the National Tobacco Control Act in Nigeria, Health Policy and Planning 36 (4): 484–492. https://doi.org/10.1093/heapol/czaa175](https://doi.org/10.1093/heapol/czaa175) | Nigeria | Fragile (LMIC) | Policy analysis |
| 161 | Diet | Kathrin Lauber, Harry Rutter, and Anna B. Gilmore. 2021. “Big Food and the World Health Organization: A Qualitative Study of Industry Attempts to Influence Global-Level Non-Communicable Disease Policy.” *BMJ Global Health* 6: e005216. | Global | Global | Qualitative method |
| 162 | Diet | [Norah Campbell, Melissa Mialon, Kathryn Reilly, Sarah Browne, Francis M. Finucane. 2020. “How Are Frames Generated? Insights from the Industry Lobby Against the Sugar Tax in Ireland.” Social Science and Medicine 264: 113215. https://doi.org/10.1016/j.socscimed.2020.113215](https://doi.org/10.1016/j.socscimed.2020.113215) | Ireland | Non fragile (HIC) | Document analysis |
| 163 | Diet | [Milkah N. Wanjohi, Ann Marie Thow, Safura Abdool Karim, Gershim Asiki, Agnes Erzse, Shukri F. Mohamed, Hermann Pythagore Pierre Donfouet, Pamela A. Juma, and Karen J Hofman. 2021. “Nutrition-Related Non-Communicable Disease and Sugar-Sweetened Beverage Policies: A Landscape Analysis in Kenya.” Global Health Action 14: 1. doi: 10.1080/16549716.2021.1902659](https://doi.org/10.1080/16549716.2021.1902659) | Kenya | Non-fragile (MIC) | Case study |
| 164 | Diet | [Angela Carriedo, Adam D. Koon, Luis Manuel Encarnación, Kelley Lee, Richard Smith, and Helen Walls. 2021. “The Political Economy of Sugar-Sweetened Beverage Taxation in Latin America: Lessons from Mexico, Chile and Colombia.” Global Health 17: 5. https://doi.org/10.1186/s12992-020-00656-2](https://doi.org/10.1186/s12992-020-00656-2) | Mexico, Chile, Colombia | Non fragile (MIC) | Case study |
| 165 | Diet | [Gemma Ahaibwe, Safura Abdool Karim, Anne-Marie Thow, Agnes Erzse, and Karen Hofman. 2021. “Barriers to, and Facilitators of, the Adoption of a Sugar Sweetened Beverage Tax to Prevent Non-Communicable Diseases in Uganda: A Policy Landscape Analysis.” Global Health Action 14 (1): 1892307. https://doi.org/10.1080/16549716.2021.1892307](https://doi.org/10.1080/16549716.2021.1892307) | Uganda | Fragile (LIC) | Case study |
| 166 | Physical activity | [Bojana Klepac Pogrmilovic, Andrea Ramirez Varela, Michael Pratt, Karen Milton, Adrian Bauman, Stuart J. H. Biddle, and Zeljko Pedisic. 2020. “National Physical Activity and Sedentary Behaviour Policies in 76 Countries: Availability, Comprehensiveness, Implementation, and Effectiveness.” International Journal of Behavioral Nutrition and Physical Activity 17: 116. https://doi.org/10.1186/s12966-020-01022-6](https://doi.org/10.1186/s12966-020-01022-6) | Global | Global | Case study |
| 167 | Diet | Colin Bell, Catherine Latu, Jeremaia Coriakula, Gade Waqa, Wendy Snowdon, and Marj Moodie. 2020. “Fruit and Vegetable Import Duty Reduction in Fiji to Prevent Obesity and Non-Communicable Diseases: A Case Study.” *Public Health Nutrition* 23 (1): 181–188. doi:10.1017/S1368980019002660 | Fiji | Non fragile (MIC) | Case study |
| 168 | NCD | [Gertrude Nsorma Nyaaba, Karien Stronks, Lina Masan, Cristina Larrea- Killinger, and Charles Agyemang. 2020. “Implementing a National Non-Communicable Disease Policy in Sub-Saharan Africa: Experiences of Key Stakeholders in Ghana.” Health Policy Open 1: 100009. https://doi.org/10.1016/j.hpopen.2020.100009](https://doi.org/10.1016/j.hpopen.2020.100009) | Sub-Saharan Africa | Fragile + Non-fragile | Case study |
| 169 | Diet | [Mulenga M. Mukanu, Safura Abdool Karim, Karen Hofman, Agnes Erzse, and Anne-Marie Thow. 2021. “Nutrition Related Non-Communicable Diseases and Sugar Sweetened Beverage Policies: A Landscape Analysis in Zambia.” Global Health Action 14: 1. doi: 10.1080/16549716.2021.1872172](https://doi.org/10.1080/16549716.2021.1872172) | Zambia | Non-fragile (MIC) | Case study |
| 170 | Primary care | [Luis Orlando Perez. 2013. Uruguay–UY Non Communicable Diseases Prevention Project : P050716–Implementation Status Results Report : Sequence 10 (). Washington, DC: World Bank Group. http://documents.worldbank.org/curated/en/841071468130210742/Uruguay-UY-Non-Communicable-Diseases-Prevention-Project-P050716-Implementation-Status-Results-Report-Sequence-10](http://documents.worldbank.org/curated/en/841071468130210742/Uruguay-UY-Non-Communicable-Diseases-Prevention-Project-P050716-Implementation-Status-Results-Report-Sequence-10) | Uruguay | Non-fragile (MIC) | Case study |
| 171 | Physical activity | Regional Committee for Africa, 70. ‎2020‎. Framework for the Implementation of the Global Action Plan on Physical Activity 2018–2030 in the WHO African Region: Report of the Secretariat. Regional Office for Africa, World Health Organization, Brazzaville, Republic of Congo. https://apps.who.int/iris/handle/10665/333737 | African Region | Fragile (+ Non-fragile | Report |
| 172 | NCD | [Sophie Witter, Guanyang Zou, Karin Diaconu, Reynold G. B. Senesi, Ayesha Idriss, John Walley, and Haja Ramatulai Wurie. 2020. “Opportunities and Challenges for Delivering Non-Communicable Disease Management and Services in Fragile and Post-Conflict Settings: Perceptions of Policy-Makers and Health Providers in Sierra Leone.” Conflict and Health 14: 3. https://doi.org/10.1186/s13031-019-0248-3](https://doi.org/10.1186/s13031-019-0248-3) | Sierra Leone | Fragile (LIC) | Mixed method |
| 173 | NCD surveillance | [Regional Office for the Eastern Mediterranean. ‎2020‎. Noncommunicable Diseases in the Eastern Mediterranean Region. Regional Office for the Eastern Mediterranean, World Health Organization, Cairo, Egypt. https://apps.who.int/iris/handle/10665/250371](https://apps.who.int/iris/handle/10665/250371) | Eastern Mediterranean Region | Fragile + Non-fragile | Report |
| 174 | COVID 19 and NCD | [World Health Organization. ‎2020‎. The Impact of the COVID-19 Pandemic on Noncommunicable Disease Resources and Services: Results of a Rapid Assessment. World Health Organization, Geneva. https://apps.who.int/iris/handle/10665/334136.](https://apps.who.int/iris/handle/10665/334136) | Global | Global | Mixed method |
| 175 | NCD | [Regional Office for South-East Asia. ‎2020‎. National Capacity for Prevention and Control of Non-Communicable Diseases in WHO SEAR—Results from NCD Country Capacity Survey 2019. Regional Office for South-East Asia, World Health Organization, New Delhi, India. https://apps.who.int/iris/handle/10665/334223](https://apps.who.int/iris/handle/10665/334223) | South East Asia | Non fragile | Quantitative method |
| 176 | Alcohol | [Tim Stockwell, Norman Giesbrecht, Kate Vallance, and Ashley Wettlaufer. 2021. “Government Options to Reduce the Impact of Alcohol on Human Health: Obstacles to Effective Policy Implementation.” Nutrients 13 (8): 2846. https://doi.org/10.3390/nu13082846](https://doi.org/10.3390/nu13082846) | Canada | Non fragile (HIC) | Case study |
| 177 | CVD | [World Health Organization. ‎2020. Technical Package for Cardiovascular Disease Management in Primary Health Care: Healthy-Lifestyle Counselling. World Health Organization, Geneva. https://apps.who.int/iris/handle/10665/260422.](https://apps.who.int/iris/handle/10665/260422) | Global | Global | Technical paper |
| 178 | CVD | [World Health Organization. ‎2020‎. Improving Hypertension Control in 3 Million People: Country Experiences of Programme Development and Implementation. World Health Organization, Geneva. https://apps.who.int/iris/handle/10665/336019.](https://apps.who.int/iris/handle/10665/336019) | Global | Global | Mixed method |
| 179 | CVD | [Nicole Fraser-Hurt, Shuo Zhang, Dayo Carol Obure, Leausa Take Naseri, Robert Thompsen, Victoria Ieremia-Faasili, and Athena Matalavea. 2020. Care for Hypertension and Other Chronic Conditions in Samoa: Understanding the Bottlenecks and Closing the Implementation Gaps. World Bank, Washington, DC. https://openknowledge.worldbank.org/handle/10986/33256](https://openknowledge.worldbank.org/handle/10986/33256) | Samoa | Fragile (MIC) [not for OECD] | Mixed method |
| 180 | CVD | [Izabella Uchmanowicz, Arno Hoes, Joep Perk, Gabrielle McKee, Margrét Hrönn Svavarsdóttir, Katarzyna Czerwińska-Jelonkiewicz, Arne Janssen, Anna Oleksiak, Paul Dendale, Ian M Graham. 2021. “Optimising Implementation of European Guidelines on Cardiovascular Disease Prevention in Clinical Practice: What Is Needed?” European Journal of Preventive Cardiology 28 (4): 426–431. https://doi.org/10.1177/2047487320926776](https://doi.org/10.1177/2047487320926776) | Europe | Non fragile (HIC) | Qualitative method |
| 181 | CVD | Dylan Collins, Tiina Laatikainen, and Jill Farrington. 2020. “Implementing Essential Interventions for Cardiovascular Disease Risk Management in Primary Healthcare: Lessons from Eastern Europe and Central Asia. *BMJ Global Health* 5: e002111. | Eastern Europe and Central Asia | Non fragile | Qualitative method |
| 182 | PC | [Tiina Laatikainen, Laura Inglin, Dylan Collins, Angela Ciobanu, Ghenadie Curocichin, Virginia Salaru, Tatiana Zatic, Angela Anisei, Diana Chiosa, Maria Munteanu, Zinaida Alexa, and Jill Farrington. 2020. “Implementing Package of Essential Non-Communicable Disease Interventions in the Republic of Moldova” European Journal of Public Health 30 (6): 1146–1151, https://doi.org/10.1093/eurpub/ckaa037](https://doi.org/10.1093/eurpub/ckaa037) | Moldova | Non fragile (MIC) | Mixed method |
| 183 | HPV | [Kelias Phiri Msyamboza, Beatrice Matanje Mwagomba, Moussa Valle, Hastings Chiumia, and Twambilire Phiri. 2017. “Implementation of a Human Papillomavirus Vaccination Demonstration Project in Malawi: Successes and Challenges.” BMC Public Health 17: 599. https://doi.org/10.1186/s12889-017-4526-y](https://doi.org/10.1186/s12889-017-4526-y) | Malawi | Non fragile (MIC) | Mixed method |
| 184 | HPV | [Ngoc-Ha Nguyen-Huu, Nathalie Thilly, Tarik Derrough, Emmanouela Sdona, Frédérique Claudot, Céline Pulcini, Nelly Agrinier HPV Policy working group. 2020. “Human Papillomavirus Vaccination Coverage, Policies, and Practical Implementation Across Europe.” Vaccine 38 (6): 1315–1331. https://doi.org/10.1016/j.vaccine.2019.11.081](https://doi.org/10.1016/j.vaccine.2019.11.081) | Europe | Non fragile (HIC) | Mixed method |
| 185 | HPV | [Caroline Soi, Joseph B. Babigumira, Baltazar Chilundo, Vasco Muchanga, Luisa Matsinhe, Sarah Gimbel, Orvalho Augusto,and Kenneth Sherr. 2019. “Implementation Strategy and Cost of Mozambique’s HPV Vaccine Demonstration Project.” BMC Public Health 19: 1406. https://doi.org/10.1186/s12889-019-7793-y](https://doi.org/10.1186/s12889-019-7793-y) | Mozambique | Fragile (LIC) | Mixed method |
