# Supplementary material for: Non-communicable disease policy implementation in Libya: A mixed methods assessment
Source: PLOS Glob Public Health. 2022 Nov 10;2(11):e0000615. doi: 10.1371/journal.pgph.0000615 (PMC10021530; doi:10.1371/journal.pgph.0000615)
Supplement: Attachment — Submitted filename: Libya Best Buy manuscript_R2R.docx [file pgph.0000615.s004.docx]

**Response to reviewer comments**

PGPH-D-22-00134
Non-Communicable Disease Policy Implementation in Libya: a mixed methods assessment

**Journal Requirements:**

1. Please include a complete copy of PLOS’ questionnaire on inclusivity in global research in your revised manuscript. Our policy for research in this area aims to improve transparency in the reporting of research performed outside of researchers’ own country or community. The policy applies to researchers who have travelled to a different country to conduct research, research with Indigenous populations or their lands, and research on cultural artefacts. The questionnaire can also be requested at the journal’s discretion for any other submissions, even if these conditions are not met.  Please find more information on the policy and a link to download a blank copy of the questionnaire here: <https://journals.plos.org/globalpublichealth/s/best-practices-in-research-reporting>. Please upload a completed version of your questionnaire as Supporting Information when you resubmit your manuscript.

- We have completed this questionnaire and uploaded it with the supporting information.

2. Please send a completed 'Competing Interests' statement, including any COIs declared by your co-authors. If you have no competing interests to declare, please state "The authors have declared that no competing interests exist". Otherwise please declare all competing interests beginning with the statement "I have read the journal's policy and the authors of this manuscript have the following competing interests:"

- We have updated this field on the submission system. It now reads:

“I have read the journal's policy and the authors of this manuscript have the following competing interests: Mohamed Aghilla and Haider M El Saeh hold senior positions in the Libyan National Centre for Disease Control.”

If you require us to fill in a specific form/statement then please let us know where to find the template and we will happily do this. Thanks.

3. Please amend your detailed Financial Disclosure statement. This is published with the article. It must therefore be completed in full sentences and contain the exact wording you wish to be published.

a. Please clarify all sources of funding (financial or material support) for your study. List the grants (with grant number) or organizations (with url) that supported your study, including funding received from your institution.

- This study was fully funded by the Middle East and North Africa (MENA) Transition Fund managed by the World Bank and implemented/conducted in collaboration with the Libyan Ministry. We have added the urls for the World Bank and Libyan MoH.

b. State the initials, alongside each funding source, of each author to receive each grant.

- No research grants or individual funding was received.

c. State what role the funders took in the study. If the funders had no role in your study, please state: “The funders had no role in study design, data collection and analysis, decision to publish, or preparation of the manuscript.”

- Representatives from the Ministry of Health contributed to the study design, analysis, and writeup.

The final statement reads:

*This project was wholly funded by the Libyan Ministry of Health (http://seha.ly/en/). The World Bank specialist team was engaged under a Reimbursable Advisory Services instrument. Representatives from the Ministry of Health contributed to the study design, analysis, and writeup.*

4. Please provide separate figure files in .tif or .eps format and removed from the manuscript file.

- We have produced separate figure files as per journal guidance.

5. We have noticed that you have uploaded Supporting Information files, but you have not included a list of legends. Please add a full list of legends for your Supporting Information files after the references list.

 - We moved the ‘supporting information’ section to the correct position and added a full list of legends:

**Supporting Information**

- Appendix
- Figure 1: PRISMA Flow Diagram
- Figure 2. Conceptual analytic framework developed to guide analysis and synthesis
- PLOS Inclusivity questionnaire

**Reviewers' comments:**
Thanks for the opportunity of reviewing this interesting, and on the whole, well written manuscript.

The manuscript does not have page numbers (or line numbers) which is an inconvenience for reviewing. Please make sure that you've complied with the instructions for authors found at <https://journals.plos.org/globalpublichealth/s/submission-guidelines>

- Apologies for the inconvenience. We have now added page and line numbers. We have also reviewed the full submission guidelines and reformatted the paper appropriately.

Please also conform to reporting guidelines for qualitative research, for example <https://www.equator-network.org/reporting-guidelines/coreq/>

- We have revised our manuscript to report our qualitative methods and findings in accordance with the 32-item COREQ checklist as helpfully suggested by the reviewer.

There is a grammatical error in the sentence - "We developed a bespoke conceptual analytic framework to synthesise and analysis of our findings"

- We have changed ‘analysis of’ to ‘analyse’

There is a "t" missing from "brought" in the sentence "Multisectoral collaboration was central to progressing PA policies in many settings, with successes seen when governments, NGOs, academia, transport, urban planners, and other stakeholders involved in PA promotion were brough together around shared policy goals."

- We have added the missing ‘t’

Some content in the methods should be put in the results instead - e.g. the conceptual framework that guided ongoing analysis, this is itself part of the findings of this study.

- We had initially placed the framework in the methods to help with sequencing and flow of the paper, however the reviewer is correct. We have now moved this section to the results.

SR-6% of studies found were from the Eastern Mediterranean, not 0.6% as stated

- We have corrected this typo.

Just because alcohol is illegal does not mean it is not consumed. I think that this needs to be more fully discussed in the manuscript.

- This is a good point and we did ask about alcohol in the interviews. We have updated the text to add additional information:
- Libya has fully implemented all Best Buy alcohol policies. Whilst our systematic review suggested that diet and alcohol share similar challenges in many settings, this is not the case in Libya – one of the few countries where alcohol is banned. Interviewees acknowledged that just because alcohol is banned does not mean that it is not consumed. However, WHO surverys conducted in 2008 and 2009 indicate that the bans are strictly enforced, domestic production and imports are negligible, and <1% of the population consumes alcohol. As such, alcohol is not perceived to be a legitimate risk factor of concern.

I find "The only material barrier is prime ministerial bandwidth and attention" a very interesting sentence. Firstly, "bandwidth" is a very informal term when used in this context - what exactly does it mean? This feels more like editorializing than scientific writing. Also - as an outsider to the context, this seems to be quite a political statement, perhaps you could comment on this?

- This was a very poorly worded sentence and it has been removed.

Our interviewees noted that health policy reform would not happen without backing at the highest levels of government. We have clarified this in the text, and note that the theme is picked up again in the ‘targets, data collection and plans’ section, the tobacco, section, and the diet section, where one of the quotes specifically singles out the Prime Minister. The synthesis section also calls out the need for high-level political buy-in. We have rephrased the conclusion:

“The main barrier to action seems to be the dependence on very high-level political buy-in. Many of the interviewees noted that progress is predicated on Prime Ministerial approval, however, the head of state has a large number of competing priorities, many of which appear more urgent than NCD risk factor control. A further proposed issue is that the current Prime Minister does not feel able to introduce health policy reforms until the new elections deliver a democratic mandate to act.”

Same comment for "Until Libya elects a prime minister who is willing to green-light nascent policy developments, allocate hypothecated resources, and engender multisectoral collaboration, our research suggests that progress is very unlikely" - this is apparently a very political comment, please provide a clearer justification for this.

- Whilst our data clearly suggested that very high-level approval is a *sine qua non* for progress, we fully agree that the wording of this sentence was injudicious We have rephrased it:

“Our review and interviews highlight the importance of high-level political leadership for NCD policy implementation. It is clear that the role of the head of the Government is crucial for progressing nascent policy developments including to allocate hypothecated resources and engender multisectoral collaboration. The backdrop of conflict, weak governance, competing health issues (including COVID-19) and a fractured health system was found to be an important limitation in the global literature, and these issues were also felt to inhibit action in a range of areas by Libyan interviewees. These contextual issues were believed to draw legislative and fiscal attention away from NCDs as well as undermine national data collection, enforcement, and harmonised health service delivery of primary care guidelines and therapies. . On one level this is understandable, however NCDs kill far more Libyans than conflict every year, and risk factor prevalence is alarmingly high. Sadly, there is little that health advocates can do to influence these macro determinants.”

It's not clear to me why your policy recommendations come before the discussion section.

- We originally placed them in the findings section due to the nature of the project. The Libyan Ministry of Health commissioned this study in order to produce a set of recommendations. As such, they were our main output. Upon reflection, we agree with the reviewer that in this academic journal article the policy recommendations would be better placed in the discussion. We have moved them.
